# Global analysis of genetic circuitry and adaptive mechanisms enabling resistance to the azole antifungal drugs

**DOI:** 10.1371/journal.pgen.1007319

**Published:** 2018-04-27

**Authors:** Harley O’Connor Mount, Nicole M. Revie, Robert T. Todd, Kaitlin Anstett, Cathy Collins, Michael Costanzo, Charles Boone, Nicole Robbins, Anna Selmecki, Leah E. Cowen

**Affiliations:** 1 Department of Molecular Genetics, University of Toronto, Toronto, Ontario, Canada; 2 Department of Medical Microbiology and Immunology, Creighton University School of Medicine, Omaha, Nebraska, United States of America; 3 Donnelly Centre for Cellular and Biomolecular Research, University of Toronto, Toronto, Ontario, Canada; Carnegie Mellon University, UNITED STATES

## Abstract

Invasive fungal infections caused by the pathogen *Candida albicans* have transitioned from a rare curiosity to a major cause of human mortality. This is in part due to the emergence of resistance to the limited number of antifungals available to treat fungal infections. Azoles function by targeting the biosynthesis of ergosterol, a key component of the fungal cell membrane. Loss-of-function mutations in the ergosterol biosynthetic gene *ERG3* mitigate azole toxicity and enable resistance that depends upon fungal stress responses. Here, we performed a genome-wide synthetic genetic array screen in *Saccharomyces cerevisiae* to map *ERG3* genetic interactors and uncover novel circuitry important for azole resistance. We identified nine genes that enabled *erg3*-mediated azole resistance in the model yeast and found that only two of these genes had a conserved impact on resistance in *C*. *albicans*. Further, we screened a *C*. *albicans* homozygous deletion mutant library and identified 13 genes for which deletion enhances azole susceptibility. Two of the genes, *RGD1* and *PEP8*, were also important for azole resistance acquired by diverse mechanisms. We discovered that loss of function of retrograde transport protein Pep8 overwhelms the functional capacity of the stress response regulator calcineurin, thereby abrogating azole resistance. To identify the mechanism through which the GTPase activator protein Rgd1 enables azole resistance, we selected for mutations that restore resistance in strains lacking Rgd1. Whole genome sequencing uncovered parallel adaptive mechanisms involving amplification of both chromosome 7 and a large segment of chromosome 3. Overexpression of a transporter gene on the right portion of chromosome 3, *NPR2*, was sufficient to enable azole resistance in the absence of Rgd1. Thus, we establish a novel mechanism of adaptation to drug-induced stress, define genetic circuitry underpinning azole resistance, and illustrate divergence in resistance circuitry over evolutionary time.

## Author summary

Fungal infections caused by the pathogen *Candida albicans* pose a serious threat to human health. Treating these infections relies heavily on the azole antifungals, however, resistance to these drugs develops readily demanding novel therapeutic strategies. We performed large-scale systematic screens in both *C*. *albicans* and the model yeast *Saccharomyces cerevisiae* to identify genes that enable azole resistance. Our genome-wide screen in *S*. *cerevisiae* identified nine determinants of azole resistance, only two of which were important for resistance in *C*. *albicans*. Our screen of *C*. *albicans* mutants identified 13 genes for which deletion enhances susceptibility to azoles, including *RGD1* and *PEP8*. We found that loss of Pep8 overwhelms the functional capacity of a key stress response regulator, calcineurin. In contrast, amplification of chromosome 7 and the right portion of chromosome 3 can restore resistance in strains lacking Rgd1, suggesting that Rgd1 may enable azole resistance by inducing genes in these amplified regions. Specifically, overexpression of a gene involved in transport on chromosome 3, *NPR2*, was sufficient to restore azole resistance in the absence of Rgd1. Thus, we establish novel circuitry important for antifungal drug resistance, and uncover adaptive mechanisms involving genomic plasticity that occur in response to drug induced stress.

## Introduction

The evolution of drug resistance in fungal pathogens poses grave concern given the limited number of antifungal drugs currently available to treat systemic infections. These infections have increased in prevalence in recent years and are now a major cause of human mortality worldwide [1], with the most vulnerable populations being those with suppressed immune function due to infection with HIV or immunosuppressive treatments for cancer or organ transplantation. *Candida albicans* is the most prevalent opportunistic human fungal pathogen and the fourth leading cause of iatrogenic bloodstream infections [2, 3]. It can colonize almost every niche in the human body and is a commensal of the mucosal microbiota, where maintenance of a stable host-fungus relationship is crucial for avoiding disease [4, 5]. Impaired host immunity enhances risks of deadly disseminated infection, with systemic candidiasis associated with mortality rates of 40% despite state-of-the-art antifungal therapies [1–3]. With the increasing prevalence of immunocompromised individuals, and the spread of drug-resistant fungal infections, there is a dire need for novel antifungal treatment options.

Currently, the most widely prescribed antifungal class is the azoles. Fluconazole has dominated this class in the clinic due to its desirable pharmacokinetics, and efficacy within the host [6, 7]. The azoles target the ergosterol biosynthetic enzyme lanosterol-14α-demethylase, Erg11, and inhibit its activity by binding to the iron heme group of its active site [6, 7]. This leads to a block in the production of ergosterol and the production of a toxic sterol intermediate by the ergosterol biosynthetic enzyme Δ-5,6-desaturase, Erg3 [6–8]. Accumulation of this toxic sterol coupled with the depletion of ergosterol destabilizes the cellular membrane, halting fungal growth and imposing a severe membrane stress upon the cell [6, 8]. The major liability of azoles is their fungistatic activity, merely inhibiting growth of the fungal population as opposed to killing the fungal cell [6, 7]. Long-term and prophylactic azole therapy coupled with a fungistatic mechanism of action generates a strong selective pressure for the evolution of azole-resistance, and azole-resistant *Candida* species have been recognized as a severe threat to public health [9].

Canonical molecular mechanisms of resistance to the azoles have been well-characterized in both laboratory-derived resistant strains, as well as clinical isolates. These include overexpression and/or alteration of the drug target Erg11, as well as overexpression of several multidrug efflux transporters, including Cdr1, Cdr2, and Mdr1 [6, 8]. Fungi exposed to fluconazole also exhibit high levels of aneuploidy, with a segmental aneuploidy involving an isochromosome of the left arm of chromosome 5 flanking a centromere (i(5L)) frequently observed in fluconazole-resistant isolates [10–12]. *C*. *albicans* also possesses numerous stress response pathways that enable survival during exposure to diverse stresses in the host, including the stress induced by antifungal drugs. Stress response pathways are crucial for basal tolerance of antifungals, as well as for resistance acquired by diverse mechanisms, including loss-of-function mutations in the ergosterol biosynthesis gene, *ERG3* [8, 13, 14]. Key stress response regulators that are crucial for *erg3-*mediated resistance include the protein phosphatase calcineurin [14, 15], protein kinase C Pkc1 [16], and molecular chaperone Hsp90 [14, 15]. The genetic circuitry through which these regulators enable resistance remains largely elusive, and it is likely that other key regulators remain to be identified. Loss of function of *erg3* provides a powerful context for defining circuitry enabling antifungal drug resistance given that *erg3* loss of function has been identified in clinical isolates [13, 17], and that key regulators of *erg3*-mediated azole resistance are also required for resistance acquired by diverse mechanisms in the host [15, 16, 18].

Systematic analysis of circuitry enabling cellular responses to drugs can now be performed with an unprecedented level of resolution by leveraging high throughput genomic analyses to define mutants with enhanced susceptibility to a drug. Such approaches have been pioneered with the model yeast *Saccharomyces cerevisiae* for which facile genetics enables a multitude of approaches to map gene function and define functional relationships in the cell [19, 20]. One approach that has yet to be applied to drug resistance, is based on synthetic genetic array (SGA) methodology, which enables the systematic mapping of synthetic genetic interactions by engineering high-density arrays of double gene deletion mutants [21–24]. Genetic interactions manifest as unexpected double mutant phenotypes and are typically assessed by quantitative measurement of fitness or morphology [20, 25], with the classic example being synthetic lethal interactions. The principle of synthetic lethality provides a rational approach to dramatically expand the target space for antimicrobial discovery based on identifying combinations of agents that target members of synthetic lethal pairs [26]. For example, aggravating genetic interaction partners of *ERG3* may represent novel drug targets for combination therapies with azoles to treat fungal infections, as in principle they should mitigate the emergence of resistance through this mechanism. Although combination therapies are the standard for treating infections such as HIV [27], malaria [28], and tuberculosis [29], this approach has remained largely unexplored for treating fungal infections. Identifying targets for combination therapy in fungal pathogens such as *C*. *albicans* is hindered by the lack of a defined meiotic cycle. An alternative strategy is to use *S*. *cerevisiae* as a model system, which is justified based on the conservation of key cellular regulators between the species [30], including the key drug resistance determinants Hsp90, calcineurin, and Pkc1 [14–16].

In this manuscript, we mapped genetic interactors of *ERG3* in *S*. *cerevisiae* in order to uncover novel genetic circuitry important for azole resistance. By screening 4,308 double mutants for growth in the presence of miconazole, we identified nine genes that enable *erg3-*mediated resistance in the model yeast. Only two of these genes had a conserved impact on *erg3-*mediated azole resistance in the fungal pathogen *C*. *albicans*, highlighting substantial divergence in the circuitry orchestrating response to drug-induced stress between the species. As an alternative approach to identify cellular responses to azoles in *C*. *albicans*, we screened a *C*. *albicans* homozygous deletion library with 1,152 mutants covering 674 genes spanning ~11% of the genome. We identified 13 *C*. *albicans* genes for which deletion enhances susceptibility to azoles. Two of the genes, *RGD1* and *PEP8*, were also important for azole resistance acquired by diverse mechanisms. We discovered that loss of function of Pep8 overwhelms the functional capacity of calcineurin in response to azole stress, thereby compromising cellular responses to membrane damage and abrogating azole resistance. In contrast, loss of function of Rgd1 did not impair canonical resistance mechanisms or stress response pathways. To identify the mechanism by which Rgd1 enables azole resistance, we selected for mutations that restore resistance in strains lacking Rgd1. Whole genome sequencing of four independent lineages uncovered parallel adaptive mechanisms involving amplification of chromosome 7 and a large segment of chromosome 3. Additional mechanistic studies demonstrated that upregulation of a transporter gene on the right arm of chromosome 3, *NPR2*, was sufficient to confer azole resistance in strains lacking Rgd1. Thus, we define complex genetic circuitry underpinning azole resistance and establish a novel mechanism through which genomic plasticity enables adaptation to drug-induced stress, with broad implications for the development of strategies to combat drug-resistant fungal infections.

## Results

### Identifying *S*. *cerevisiae ERG3* genetic interactors under basal conditions, and in response to drug-induced stress

**Fig 1:**
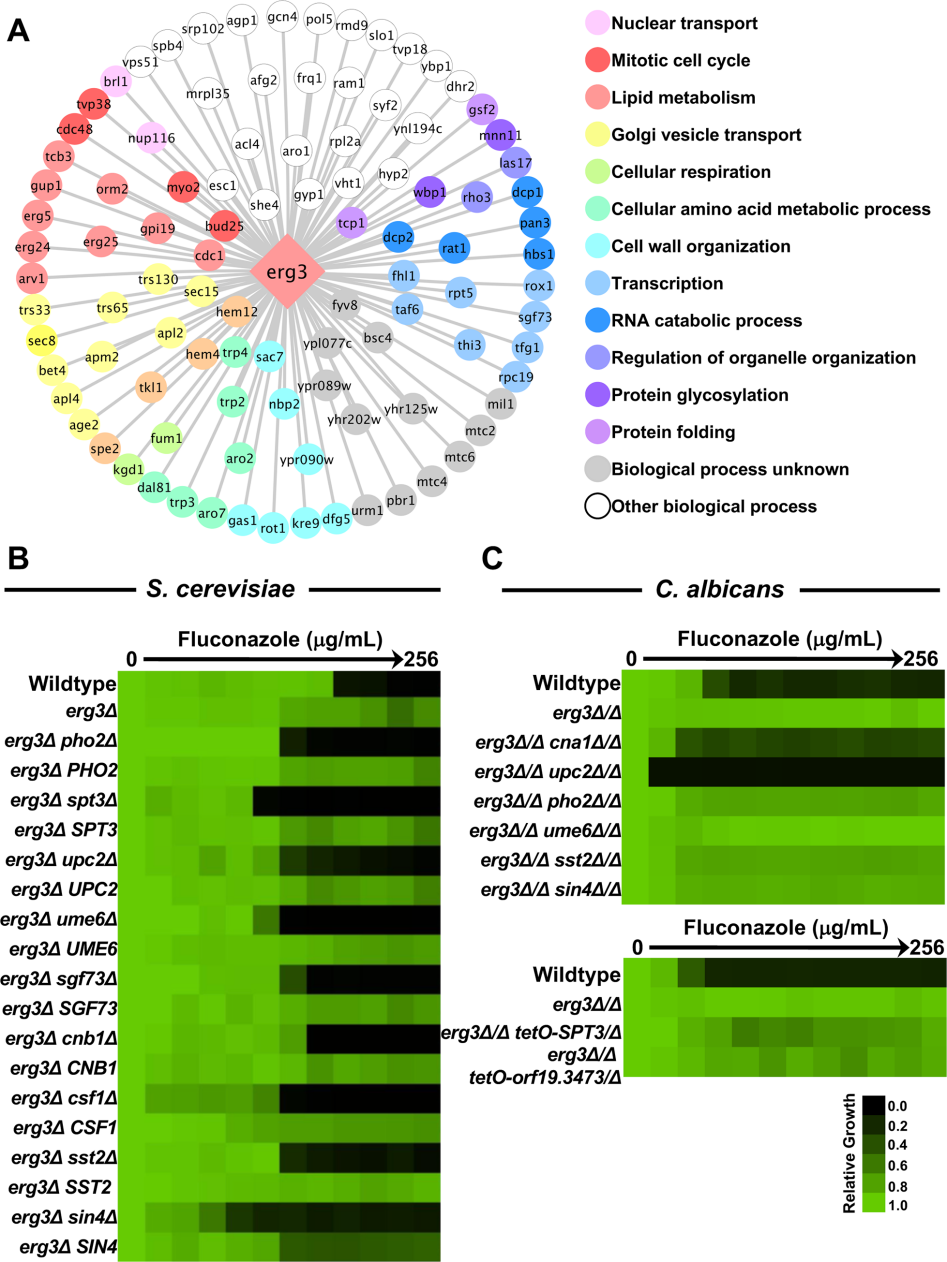
The *ERG3* genetic interaction network in *S*. *cerevisiae* uncovers species-specific circuitry important for azole resistance. **A)** Genetic nodes highlighting the negative genetic interactions with an SGA score (ε) ≤ -0.25 in YPD medium. 100 genes were identified as having a genetic interaction with *ScERG3*, and they are coloured based on their GO Slim Process category. **B)** Microbroth dilution minimum inhibitory concentration (MIC) assay for *S*. *cerevisiae ERG3* genetic interactors for which gene deletion abrogates azole resistance of the *erg3* mutant. Strains were complemented with their respective wild-type allele to verify the azole sensitivity phenotype was due to the specific gene deletion. MIC assays were conducted in YPD and growth was measured by absorbance at 600 nm after 48 hours at 30°C. Optical densities were averaged for duplicate measurements. Data was quantitatively displayed with colour using Treeview (see colour bar). **C)** MIC assay for *C*. *albicans erg3* homozygous deletion mutants that also harboured homozygous deletions of genes originally identified in *S*. *cerevisiae* as being important for *erg3-*mediated resistance. MIC assay was performed as in part B. For the bottom panel, fluconazole MIC assays were conducted in YPD medium with 1 μg/mL doxycycline to achieve transcriptional repression of the target gene in the conditional expression strains.

We leveraged the power of genome-scale synthetic genetic analysis in *S*. *cerevisiae* to define genetic interactors of a key regulator of membrane homeostasis and drug resistance, *ERG3*. By monitoring fitness in the absence and presence of azole, we aimed to define condition-specific genetic interactions, and new determinants of drug resistance. Using SGA technology, we mapped genetic interactions by generating double deletion mutants using an *erg3Δ* query strain and the non-essential gene deletion library [21]. In the absence of a genetic interaction, double-mutant fitness is a multiplicative combination of each single-mutant fitness, with genetic interactions defined as deviations from the expected double-mutant fitness [25]. These interactions are assigned an SGA score, with the magnitude of the score indicative of the strength of the genetic interaction. We identified 100 genetic interaction partners of *ScERG3* under standard laboratory conditions with an SGA score ≤ -0.25 [20], including genes involved in diverse biological processes such as lipid metabolism, Golgi vesicle transport, transcription, cellular amino acid metabolic process and cell wall organization (Fig 1A and S1 Table). Next, we compared our genetic interaction profile with that observed for other query genes, given that similarity of genetic interaction profiles reveals functional relationships between genes. As expected, the *ERG3* genetic interaction profile was significantly correlated with that observed for genes involved in ergosterol biosynthesis including *ERG6*, *ERG24*, *ERG2*, and *ERG25* (S1 Table). This analysis supports the importance of Erg3 in ergosterol biosynthesis and implicates interactions with other diverse biological processes.

In order to specifically identify genes that are required for azole resistance acquired by *ERG3* loss of function, we extended the SGA approach to screen the full collection of double mutants in the presence of a fixed concentration of the azole miconazole and monitored growth relative to the *erg3Δ* strain (S2 Table). For the double mutants identified as sensitive to miconazole in the primary screen, we performed a complementary drug susceptibility test by performing a fluconazole minimum inhibitory concentration (MIC) assay. Through this analysis we validated nine out of 14 genes for which deletion suppressed the *erg3-*mediated azole resistance phenotype and whose complementation with the wild-type allele restored *erg3*-mediated azole resistance (*PHO2*, *SPT3*, *UPC2*, *UME6*, *SGF73*, *CNB1*, *CSF1*, *SST2*, and *SIN4*) (Fig 1B). The remaining four genes (*BAP2*, *TRP4*, *MDM39* and *BRE1*) did not validate in our follow up analysis. Notably, we identified known determinants of the *erg3-*mediated resistance phenotype, including the gene encoding the catalytic subunit of calcineurin, *CNB1*, validating our approach. Thus, by leveraging SGA, we identified genome-scale functional relationships with *ERG3*, and genes important for azole resistance.

### Divergence of *ERG3* genetic interactors between fungal species

We next assessed whether the nine genes for which deletion suppressed *erg3*-mediated azole resistance in *S*. *cerevisiae* had a conserved impact on azole resistance in the fungal pathogen *C*. *albicans*. To do so, we generated homozygous deletions of each gene in a *C*. *albicans erg3Δ/erg3Δ* mutant background. Due to difficulty in constructing homozygous deletion mutants for two genes, *SPT3* and *orf19*.*3473*, we engineered conditional expression strains in which we deleted one allele and replaced the native promoter of the remaining allele with a tetracycline-repressible promoter to enable transcriptional repression by the tetracycline analog doxycycline. As expected, homozygous deletion of the gene encoding the catalytic subunit of calcineurin, *CNA1*, or a transcription factor required for the upregulation of drug efflux pumps, *UPC2*, abrogated *erg3-*mediated azole resistance of *C*. *albicans* (Fig 1C) [15, 31–33]. In contrast, deletion or transcriptional repression of the remaining six genes tested had no impact on azole resistance (Fig 1C), revealing substantial divergence in the genetic circuitry underpinning azole resistance between the two species.

### Functional genomic screening in *C*. *albicans* identifies genes required for basal tolerance to fluconazole and for *erg3-*mediated azole resistance

**Fig 2:**
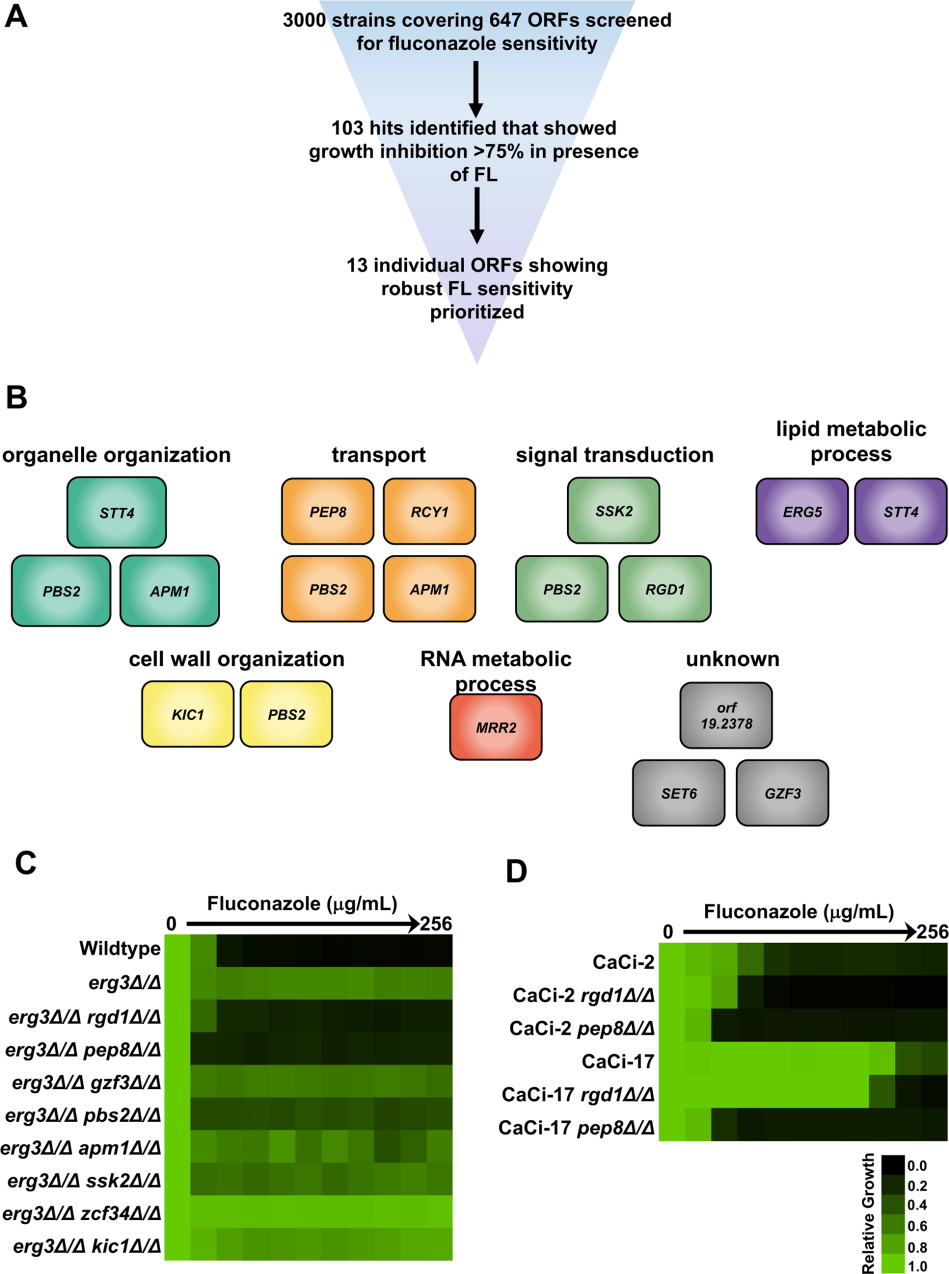
*C*. *albicans* functional genomic screen identifies novel genetic circuitry important for azole tolerance and resistance. **A)** Schematic of functional genomic screen to identify genes important for fluconazole (FL) tolerance. Thirteen genes were identified and prioritized for follow-up analysis based on the robust hypersensitivity of the corresponding deletion mutants. **B)** The 13 genes that were identified as having important roles in fluconazole tolerance, coloured based on their GO Slim Process category. Note many genes belong to multiple categories. **C)** MIC assay for *C*. *albicans erg3* homozygous deletion mutants that also harboured homozygous deletions of genes implicated in fluconazole tolerance in part B. Homozygous deletion of either *RGD1* or *PEP8* abrogates the *erg3-*mediated azole resistance phenotype. MIC assay was performed as described in Fig 1. Growth was analyzed after 24 hours (see colour bar). **D)** Homozygous deletion of either *RGD1* or *PEP8* reduces azole resistance of two resistant clinical isolates collected from an HIV patient undergoing fluconazole therapy. CaCi-2 represents an isolate collected early in treatment and CaCi-17 represents an isolate collected later in treatment. MIC assay was performed as described in part C.

Strains of *C*. *albicans* are often capable of proliferating in the presence of azoles independent of specific adaptive mutations, a phenomenon referred to as tolerance. Based on the findings that regulators of *erg3-*mediated azole resistance are often important for basal tolerance of wild-type cells to azoles, as is the case with *CNA1* and *UPC2* [15, 33], we took a complementary approach to define regulators of azole resistance. We screened a *C*. *albicans* library of homozygous deletion mutants spanning approximately 11% of the genome [34], for hypersensitivity to a sub-inhibitory concentration of fluconazole (Fig 2A). Using this approach, we identified 13 sensitive strains that had an OD_600_ < 80% that of the wild type in the presence of azole, with minimal growth defect in the absence of drug (Fig 2B): *ERG5*, *KIC1*, *PEP8*, *APM1*, *GZF3*, *STT4*, *PBS2*, *RGD1*, *RCY1*, *SSK2*, *MRR2*, *SET6*, and *orf19*.*2378*. Although five of these genes, *ERG5*, *KIC1*, *GZF3*, *PBS2*, and *MRR2*, had previously been linked to azole tolerance [35], the remaining eight genes had previously unreported roles.

To determine if the genes that influence basal tolerance also enable *erg3-*mediated azole resistance, we deleted both *ERG3* alleles in eight of the 13 homozygous deletion mutant backgrounds. The remaining five genes were not evaluated based on difficulty with mutant construction. This identified two of the eight genes as important for *erg3*-mediated azole resistance, *RGD1* and *PEP8* (Fig 2C). *RGD1* encodes a RHO-GTPase activating protein (RHO-GAP), which facilitates the GTP hydrolysis of its cognate RHO-GTPases Rho3, Rho4, and Cdc42 [36], and *PEP8* encodes a vacuolar protein component of the retromer that is essential for endosome-to-Golgi retrograde protein transport [37]. To our knowledge, neither *PEP8* nor *RGD1* have been previously implicated in azole resistance in *C*. *albicans*. We found that the *pep8* and *rgd1* homozygous deletion mutants had a sensitivity to specific cell membrane stresses, as the mutants were hypersensitive to the membrane perturbing agents SDS and terbinafine, but were not sensitive to another membrane perturbing agent amphotericin B or to cell wall stress induced by the echinocandin caspofungin (S1A Fig). Thus, our screen for genes enabling fluconazole tolerance identified two novel determinants of *erg3-*mediated azole resistance in *C*. *albicans*.

### Rgd1 and Pep8 enable azole resistance of clinical isolates harboring diverse resistance mechanisms

Although *ERG3* loss-of-function mutations are relatively common among clinical isolates [13, 38, 39], we wanted to assess whether *PEP8* and *RGD1* have an impact on azole resistance that evolved in a human host. We focused on two strains from a series of *C*. *albicans* oral isolates from an HIV-infected individual sampled over a 2-year period of treatment with fluconazole; CaCi-2 represents one of the first isolates collected over this time period and CaCi-17 is the final isolate collected [40]. Throughout the sampling period, these isolates acquired diverse mutations including point mutations in *UPC2*, *ERG11* and *TAC1*, which led to the overexpression of efflux pumps and the azole target *ERG11* [40–42]. We engineered *RGD1* and *PEP8* homozygous deletion mutants in each clinical isolate background and assessed azole susceptibility phenotypes. Homozygous deletion of either *RGD1* or *PEP8* enhanced the fluconazole susceptibility of both clinical isolates to differing extents (Fig 2D and S1B Fig). Deletion of *PEP8* had the strongest effect on resistance, reducing the fluconazole minimum inhibitory concentration by approximately four-fold in the early isolate, and 128-fold in the late clinical isolate. The enhanced susceptibility due to *RGD1* deletion was less pronounced, with an approximately two-fold effect in both the early and late clinical isolates. Thus, identifying genes important for azole tolerance and *erg3*-mediated azole resistance provides a powerful strategy to discover novel regulators of clinically-relevant azole resistance.

### Loss of function of *PEP8* impairs stress response signaling

**Fig 3:**
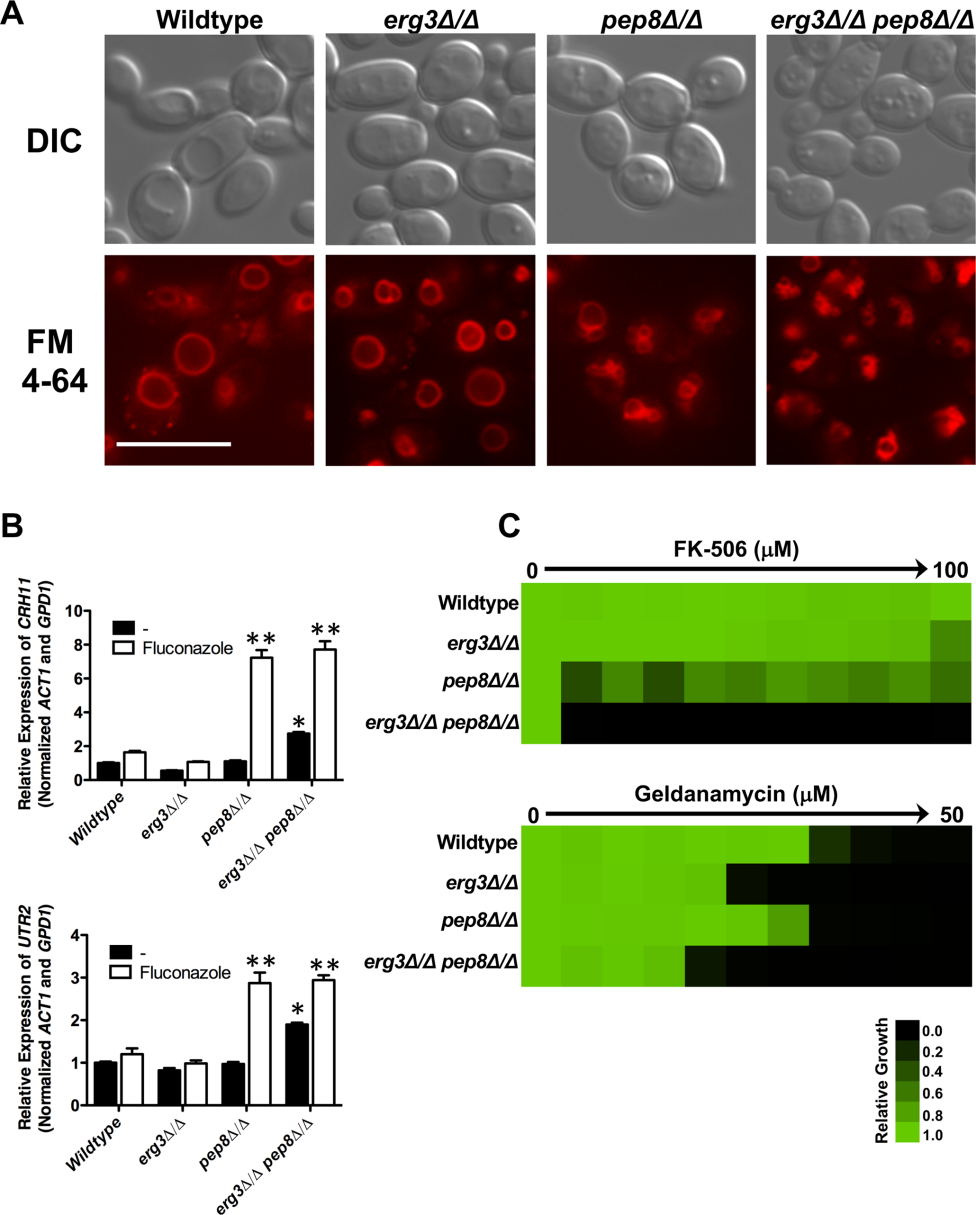
Loss of *PEP8* results in abnormal vacuolar morphology and overwhelms the functional capacity of calcineurin. A) Strains of *C*. *albicans* were grown to log-phase prior to staining with membrane dye FM4-64. Images were captured using differential interference contrast (DIC) microscopy and fluorescence microscopy with a TRITC/DsRED filter set on a Zeiss Axio Observer.Z1 (Carl Zeiss) using 100x magnification. Scale bar represents 10 μm. B) Deletion of *PEP8* increases fluconazole-induction of two calcineurin-dependent transcripts, *UTR2* and *CRH11*. Transcript levels were monitored by qRT-PCR and normalized to *ACT1* and *GPD1*. Error bars represent standard error of the mean for triplicate samples. Treatment conditions were compared using a one-way ANOVA with Bonferroni post-test. Double asterisk indicates a significant difference in transcript level relative to both wildtype with fluconazole and *erg3Δ/erg3Δ* strain plus fluconazole. (** *P<*0.01). Asterisk indicates a significant difference in transcript level relative to the wildtype and individual deletion mutants under basal conditions (* *P<*0.05). C) Deletion of *PEP8* enhances sensitivity of *C*. *albicans* strains to the calcineurin inhibitor, FK-506, as well as the Hsp90 inhibitor geldanamycin. MIC assay was performed as described in Fig 1. Growth was measured after 24 hours.

Determining the mechanisms through which novel regulators of azole resistance enable cellular responses to drug-induced stress is crucial for understanding circuitry enabling drug resistance and identifying new targets for intervention. In the context of *PEP8*, members of the retrograde transport pathway have been implicated in facilitating tolerance to a wide variety of cellular stresses including alkalinity, SDS, and high concentrations of Ca^2+^ in *C*. *albicans* [43]. Disruption of retrograde trafficking results in perturbed vacuolar morphology, and increased levels of calcineurin-dependent transcripts, which are necessary to survive the stress imposed by a retrograde transport defect [43]. We hypothesized that the *erg3Δ/erg3Δ* *pep8Δ/pep8Δ* double deletion mutant may experience a state of cellular stress that overwhelms the functional capacity of calcineurin upon fluconazole exposure. To test this, we first verified that the *pep8Δ/pep8Δ* mutant displayed perturbed vacuolar morphology, as visualized by DIC microscopy and FM4-64 staining (Fig 3A and S2 Fig). Indeed, vacuoles in *pep8Δ/pep8Δ* mutant were less well-defined compared to the wild type or *erg3Δ/erg3Δ* mutant. This effect was exacerbated in an *erg3Δ/erg3Δ* *pep8Δ/pep8Δ* double mutant, with vacuoles almost completely fragmented (Fig 3A and S2A Fig). We also observed fragmented vacuoles when *PEP8* was deleted from clinical isolates CaCi-2 and CaCi-17, highlighting this was not specific to the *erg3* mutant background (S2B Fig). Next, we tested whether deletion of *PEP8* resulted in an increase in calcineurin-dependent transcripts. Using quantitative RT-PCR, we observed that the *pep8Δ/pep8Δ* and *erg3Δ/erg3Δ* *pep8Δ/pep8Δ* mutants had significantly elevated levels of the calcineurin-dependent transcripts *CRH11* and *UTR2* relative to the wild type and *erg3Δ/erg3Δ* parental strains in the presence of fluconazole, consistent with elevated calcineurin activity (Fig 3B). *CRH11* and *UTR2* expression levels were also increased in the *erg3Δ/erg3Δ* *pep8Δ/pep8Δ* double mutant in the absence of fluconazole relative to the individual deletion mutants, suggesting that the protein phosphatase was hyperactivated under basal conditions in this double mutant (Fig 3B). Notably, in an *erg3* mutant, constitutive calcineurin activation does not abrogate azole resistance as homozygous deletion of *ERG3* in a strain lacking the autoinhibitory domain of calcineurin [44], did not lead to an enhanced susceptibility to azoles (S3A Fig). This highlights that constitutive activation of calcineurin alone is not sufficient to abrogate *erg3*-mediated resistance implicating additional cellular consequences upon loss of function of Pep8. Collectively, these results confirm that deletion of *PEP8* results in perturbed vacuolar morphology and increased levels of calcineurin effector genes, and these effects are exacerbated in combination with deletion of *ERG3* under basal conditions.

Our findings are consistent with the model that deletion of both *ERG3* and *PEP8* results in an increase in cellular stress that overwhelms the functional capacity of calcineurin. An additional prediction of the model is that *erg3Δ/erg3Δ* *pep8Δ/pep8Δ* double mutants would be hypersensitive to calcineurin inhibition. We tested the sensitivity of wild type, *erg3Δ/erg3Δ*, *pep8Δ/pep8Δ*, and *erg3Δ/erg3Δ* *pep8Δ/pep8Δ* strains to the calcineurin inhibitor FK-506. Although the *pep8Δ/pep8Δ* mutant displayed some sensitivity to FK-506 relative to the wild-type strain, the *erg3Δ/erg3Δ* *pep8Δ/pep8Δ* double mutant showed striking hypersensitivity to FK506 relative to either single mutant or the parental strain (Fig 3C and S3B Fig). Yet another prediction of our model is that the *erg3Δ/Δ* *pep8Δ/Δ* double mutant would be hypersensitive to Hsp90 inhibition, given that inhibition of Hsp90 destabilizes the catalytic subunit of calcineurin and impairs calcineurin activation in response to stress [18]. As predicted, the *pep8Δ/pep8Δ* and *erg3Δ/erg3Δ* mutants both displayed enhanced susceptibility to the Hsp90 inhibitor geldanamycin relative to the wild-type strain, and this sensitivity was further enhanced in the *erg3Δ/erg3Δ* *pep8Δ/pep8Δ* double mutant (Fig 3C and S3B Fig). Together, these findings support a model in which loss of Pep8 induces cellular stress which is further exacerbated by loss of Erg3, thereby overwhelming the demand for calcineurin and impairing crucial calcineurin-dependent cellular stress responses and abrogating azole resistance.

### Rgd1 enables azole resistance independent of established resistance mechanisms

We next sought to evaluate the mechanism by which Rgd1 influences azole resistance, by monitoring the impact of this RHO-GAP on known resistance circuitry. First, we quantified the expression of known resistance determinants, including key multidrug transporters and the azole target *ERG11*. In strains lacking *RGD1*, both the basal levels of *ERG11* and the induction in response to fluconazole were comparable to the wild-type strain for the *rgd1Δ/rgd1Δ* mutant and comparable to the *erg3Δ/erg3Δ* control for the *erg3Δ/erg3Δ* *rgd1Δ/rgd1Δ* double mutant (S4A Fig). Similarly, homozygous deletion of *RGD1* in either a wild type or *erg3Δ/erg3Δ* background did not lead to a decrease in expression of efflux transporters *CDR1*, *CDR2*, or *MDR1*, and in some cases actually led to an increase in expression in the presence of fluconazole (S4A Fig). Second, we tested the impact of Rgd1 on protein kinase C (PKC) signaling, given that PKC signaling enables azole resistance in *C*. *albicans* [16], and that Rgd1 has been implicated as a positive regulator of PKC signaling in *S*. *cerevisiae* and *C*. *albicans* in response to low pH stress [45–47]. We found that the terminal MAP kinase in the Pkc1 cascade, Mkc1, was phosphorylated in response to fluconazole in both wild-type and *rgd1Δ/rgd1Δ* strains (S4B Fig), suggesting that Rgd1 is not required for activation of this cascade in response to drug-induced stress. Intriguingly, Mkc1 was phosphorylated under basal conditions in both *erg3Δ/erg3Δ* and *erg3Δ/erg3Δ* *rgd1Δ/rgd1Δ* mutants, suggesting that the Pkc1 pathway is constitutively activated in strains lacking Erg3 (S4B Fig). Finally, to test if deletion of *RGD1* resulted in impaired signaling through other stress response pathways important for azole resistance, we monitored calcineurin activation by measuring the induction of the calcineurin-dependent transcript, *UTR2*. Both the basal expression and upregulation of *UTR2* in response to fluconazole were comparable between strains lacking Rgd1 and the controls (S4C Fig), suggesting that the azole sensitivity of the *erg3Δ/erg3Δ* *rgd1Δ/rgd1Δ* mutant cannot be attributed to a defect in calcineurin signaling. Thus, *RGD1* enables azole resistance independent of effects on expression of the drug target gene or activation of key stress response pathways implicated in azole resistance.

### Rgd1 enables azole resistance independent of Rho3, Rho4 or Pikα

As a complementary approach to determine the mechanism through which Rgd1 enables azole resistance, we tested whether known physical interaction partners of Rgd1 were important for this phenotype. First, we focused on Rho3 and Rho4, which are RHO-GTPases that are activated by Rgd1 in *S*. *cerevisiae* and involved in actin cytoskeleton re-organization and polarized cell growth [48]. We found that homozygous deletion of *RHO3* or *RHO4* had no impact on fluconazole susceptibility in an otherwise wild-type background or in an *erg3* mutant background (S4D Fig). Next we focused on Pik1, a phosphoinositide kinase that regulates localization of Rgd1 to the plasma membrane in *S*. *cerevisiae* [49]. As with *RHO3* and *RHO4*, we found that homozygous deletion of the *C*. *albicans* homolog of *PIK1*, *PIKα*, did not enhance azole susceptibility (S4D Fig). Thus, Rgd1 enables cellular responses to azoles independent of known interacting partners.

### Aneuploidy of chromosome 3 and 7 restores azole resistance in an *rgd1* mutant

**Fig 4:**
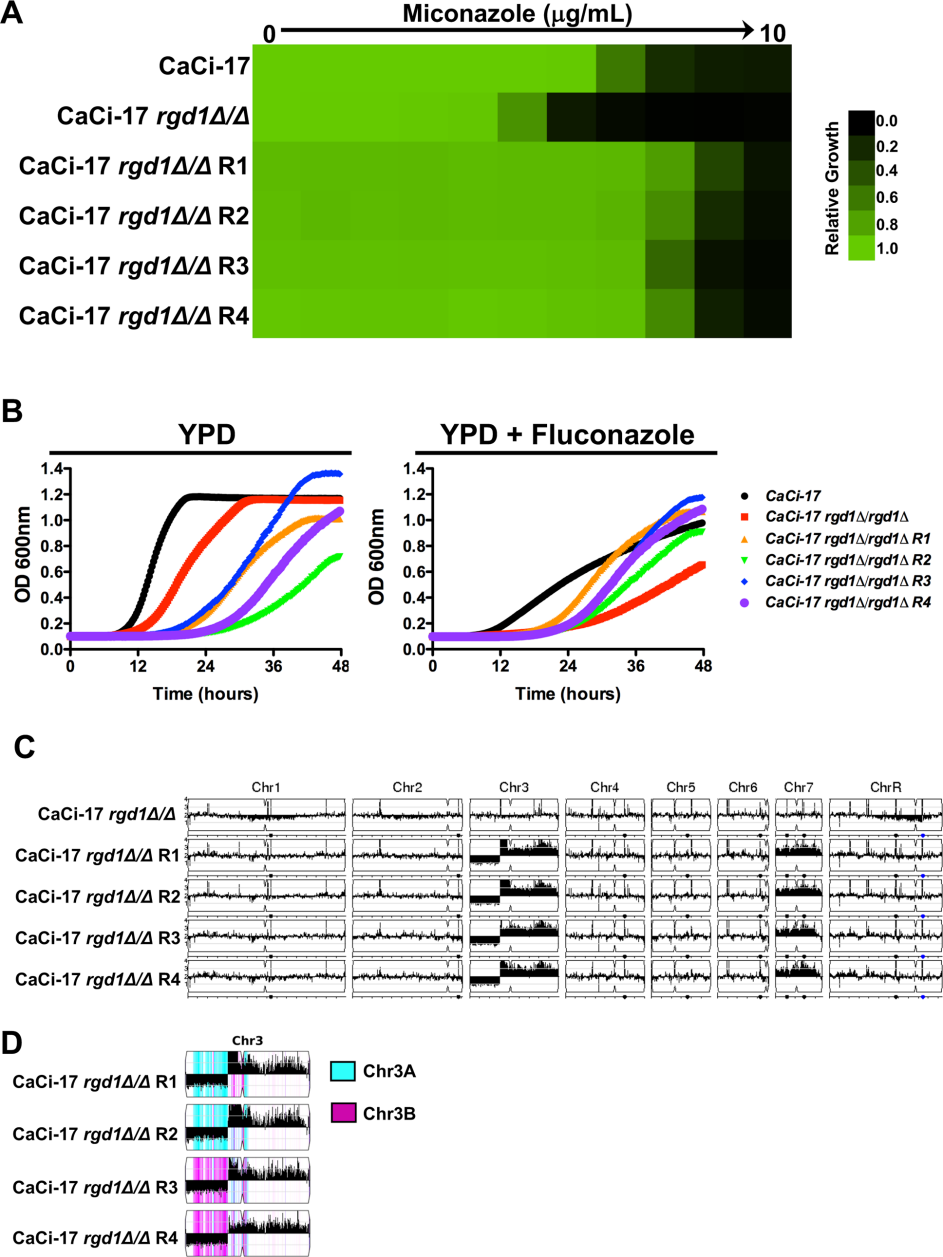
Whole genome sequencing identifies aneuploidies that are associated with the restoration of azole resistance to a *C*. *albicans* clinical isolate lacking *RGD1*. **A)** *RGD1* was deleted from a clinical isolate obtained late in treatment from a patient undergoing fluconazole therapy (CaCi-17). Resistant mutants were obtained by plating 2x10^8^ cells on YPD plates containing a high concentration of miconazole. Spontaneous mutants were selected after 5 days at 30°C. Azole resistance of the mutants was verified by MIC assay. Cells were grown as described in Fig 1. Growth was measured after 48 hours. **B)** Spontaneous azole resistant mutants show fitness defect in the absence of the selective pressure but show enhanced growth in the presence of fluconazole. Strains were grown in the absence or presence of 128 μg/mL fluconazole. Growth was assessed by OD_600_ measurements every 15 minutes for 48 hours in a Tecan plate reader. **C)** Copy number variation was analyzed using the Y_MAP_ pipeline. Amplification of chromosome 7 as well as a portion of chromosome 3 occurred in all independent spontaneous resistant mutants. **D)** Chromosome 3 haplotype map analyzed using Y_MAP_. The monosomic region of Chr3L was generated from different haplotypes in the four azole-resistant isolates: haplotype A (Cyan) in isolates R1 and R2, and haplotype B (magenta) in isolates R3 and R4.

Given that the mechanism by which Rgd1 enables azole resistance remained elusive, we turned to an unbiased approach to identify mutations that could restore azole resistance to a strain lacking Rgd1. To do so, we plated ~2x10^8^ cells on agar medium containing a high concentration of miconazole for the sensitized *erg3Δ/erg3Δ* *rgd1Δ/rgd1Δ* strain, as well as the CaCi-17 *rgd1Δ/rgd1Δ* strain. We were unable to recover resistant mutants in the *erg3Δ/erg3Δ* *rgd1Δ/rgd1Δ* background. In contrast, many resistant isolates from independent lineages were obtained from the CaCi-17 *rgd1Δ/rgd1Δ* background, each of which had enhanced azole resistance relative to the progenitor in addition to a growth defect in the absence of drug (Fig 4A and 4B). Thus, there are fitness constraints that may limit the evolution of azole resistance in the absence of Rgd1.

To identify mutations that could restore resistance in the absence of Rgd1, we performed whole genome sequencing of four azole-resistant isolates from independent selection experiments, along with the respective progenitor strain. Genome sequence analysis did not identify any single nucleotide variants or small insertions or deletions in coding regions that were common to the evolved lineages and absent from the progenitor. In contrast, we discovered several large structural changes that were common among the azole-resistant isolates, involving copy number expansions of chromosome 3 and 7 (Fig 4C). In particular, all four evolved lineages had a common breakpoint on the left arm of chromosome 3 that simultaneously resulted in a loss (monosomy) of 35% of the left portion of chromosome 3 and amplification of 65% of the right portion of chromosome 3. All four evolved clones had different levels of chromosome 3 amplification (Fig 4C), and allele ratios within the amplification indicate that these were likely independent events (Fig 4D). For example, the monosomic region of chromosome 3 was composed of haplotype A in two of the lineages (R1 and R2) and haplotype B in the other two evolved lineages (R3 and R4) (Fig 4D). Thus, dramatic structural rearrangements of chromosome 3 and 7 restore azole resistance in a clinical isolate lacking *RGD1*.

We scanned the genes within the expanded region of chromosomes 3 and 7 to identify any with annotated roles in response to azoles or membrane transport and identified several candidate resistance modifiers. For example, the amplified region of chromosome 3 contains: orf19.344, which is upregulated in azole-resistant strains and is thought to be regulated by the efflux regulator Tac1 [50, 51]; orf19.304, which is a putative transporter similar to MDR proteins [52]; and Npr2, which is a putative urea transporter [53]. Chromosome 7 harbors the cellular stress response regulator Hsp90 [15], the major facilitator transporter Flu1 with reported roles in fluconazole susceptibility [54], and other sugar transporters including Hgt12 and Hgt13. We quantified expression of these candidate genes by qRT-PCR to determine if the increased copy number was associated with increased expression and confirmed elevated transcript levels of all genes in the resistant isolates relative to the parental control (S5A Fig). A concomitant increase in resistance to the Hsp90 inhibitor geldanamycin was also observed in all four resistant isolates (S5B Fig). Finally, we monitored the accumulation of rhodamine 6G (R6G) in our parental and evolved isolates. R6G is a fluorescent substrate that is effluxed from the cell by many of the transporters that are involved in fluconazole efflux [55]. Deletion of *RGD1* from the azole-resistant isolate CaCi-17 resulted in an increased accumulation of the fluorescent substrate within the cell relative to CaCi-17 (S6 Fig), suggesting that this strain was impaired in drug efflux. However, in all four evolved lineages R6G accumulation was reduced relative to the CaCi-17 *rgd1Δ/rgd1Δ* background (S6 Fig), highlighting these strains had improved efflux relative to their fluconazole-susceptible parent. Thus, amplification of chromosome 3 and 7 enables the evolution of antifungal drug resistance, likely through the upregulation of multiple genes involved in efflux and stress response signaling.

**Fig 5:**
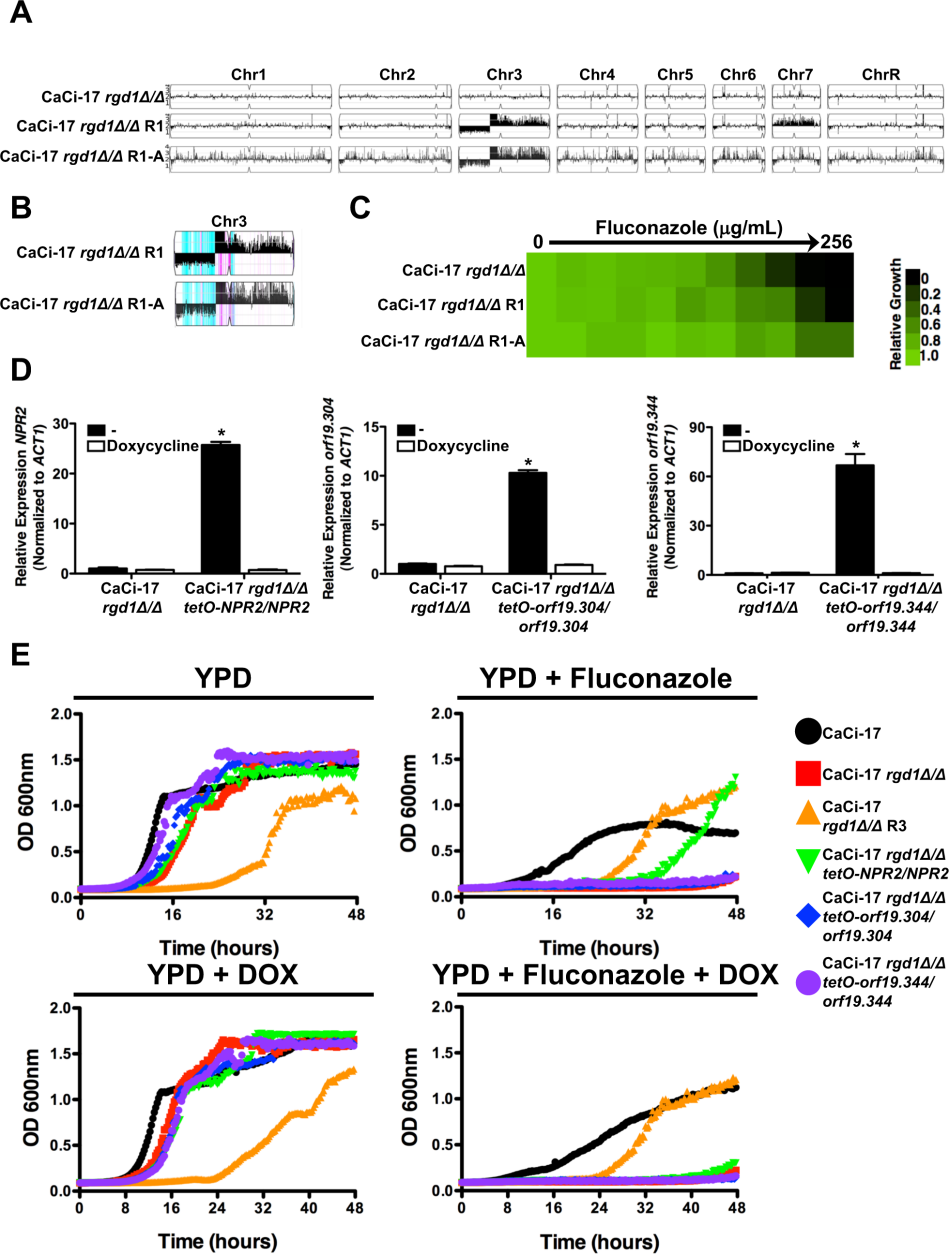
Overexpression of *NPR2*, a putative urea transporter located in the amplified portion of chromosome 3, in the evolved resistant isolates is sufficient to confer enhanced resistance to azoles in the absence of *RGD1*. **A)** The azole-resistant CaCi-17 *rgd1Δ/rgd1Δ* R1 lineage was passaged without azole and an individual colony with improved fitness in the absence of stress was selected (CaCi-17 *rgd1Δ/rgd1Δ* R1-A). Copy number variation was analyzed using the Y_MAP_ pipeline. Loss of amplification of chromosome 7 was observed, however, the unusual chromosome 3 aneuploidy was maintained. **B)** Chromosome 3 haplotype map analyzed using Y_MAP_. For the newly evolved CaCi-17 *rgd1Δ/rgd1Δ* R1-A isolate, the monosomic region of Chr3L and highly amplified region of Chr3L show similar haplotype composition as the parental CaCi-17 *rgd1Δ/rgd1Δ* R1 strain: haplotype A (Cyan), and haplotype B (magenta). **C)** Azole resistance of indicated mutants was evaluated by MIC assay. Cells were grown as described in Fig 1. Growth was measured after 24 hours and normalized to the no drug control for each strain. See colour bar. **D)** Replacing the native promoter of one allele with the strong *tetO* promoter results in elevated expression of *NPR2* (left panel), *orf19*.*304* (middle panel), and *orf19*.*344* (right panel). Addition of doxycycline reduced expression of each transcript. Transcript levels were monitored by qRT-PCR and normalized to *TEF1*. Error bars represent standard error of the mean for triplicate samples. Expression levels of evolved strains were compared to the parental strain using a one-way ANOVA with Bonferroni post-test. Asterisk indicates significant difference in transcript level relative to the parental strain in the absence of doxycycline (* *P<*0.01). **E)** Overexpression of *NRP2* results in enhanced azole susceptibility in the CaCi-17 *rgd1Δ/rgd1Δ* background. Strains were grown in the absence or presence of 128 μg/mL fluconazole, 0.05 μg/mL doxycycline (DOX), or a combination of fluconazole and DOX as indicated. Growth was assessed by OD_600_ measurements every 15 minutes for 48 hours in a Tecan plate reader.

To further define the gene(s) that enable azole resistance in the absence of *RGD1*, we plated one of our evolved resistant isolates, CaCi-17 *rgd1Δ/Δ* R1, for single colonies onto YPD agar and allowed the culture to grow in the absence of drug stress for three days. Whole genome sequencing of selected colonies identified one lineage that had lost the chromosome 7 aneuploidy but retained the unusual chromosomal 3 aneuploidy (Fig 5A and Fig 5B). This isolate retained the fluconazole resistance phenotype as evidenced by minimum inhibitory concentration assays, highlighting that the gene(s) responsible for azole resistance are likely located on chromosome 3 (Fig 5C). Based on this prediction, we focused our analysis on those genes with annotated roles in response to azoles or membrane transport that were located in the amplified region of chromosome 3. In the CaCi-17 *rgd1Δ*/*Δ* strain, we overexpressed *orf19*.*344*, *orf19*.*304* or *NPR2* by replacing the native promoter of one allele with the strong tetracycline-repressible promoter. In the absence of tetracycline or its analog doxycycline (DOX), each of these transcripts was significantly overexpressed, and expression was reduced upon growth of cells with DOX (Fig 5D). Drug susceptibility profiling demonstrated that overexpression of *NPR2* increased azole resistance relative to the CaCi-17 *rgd1Δ*/*Δ* parent (Fig 5E), confirming that overexpression of *NPR2* is sufficient to enable azole resistance in the absence of Rgd1. Notably, overexpression of *orf19*.*344* or *orf19*.*304* had no effect on azole susceptibility (Fig 5E). Thus, we implicate the putative urea transporter, Npr2, as a mediator of azole resistance in the absence of *RGD1* and identify a fascinating example of parallel evolution in which the amplification of a transporter gene on chromosome 3 is the preferred adaptive mechanism to enable azole resistance in the absence of this RHO-GTPase activating protein.

## Discussion

In this study, we uncovered novel genetic circuitry that enables resistance to the most widely deployed class of antifungal drugs, the azoles. Leveraging genome-scale synthetic genetic analysis in *S*. *cerevisiae*, we identified *ERG3* genetic interactors under basal conditions and in the presence of miconazole, nine of which proved to be determinants of azole resistance. Our analysis revealed considerable divergence in genes important for azole resistance in *C*. *albicans*, motivating an alternate approach to screen for genes that enable basal tolerance to azoles directly in this leading human fungal pathogen. A functional genomic screen of *C*. *albicans* mutants covering ~11% of the genome, identified 13 genes important for azole tolerance. Two of these genes, *RGD1* and *PEP8*, were required for azole resistance acquired by diverse mechanisms. We found that loss of Pep8 overwhelms the functional capacity of calcineurin in response to azole stress, impairing crucial responses to drug-induced cell membrane stress. In contrast, loss of Rgd1 did not impair canonical resistance mechanisms or signaling through key stress response pathways implicating novel genetic circuitry governing azole resistance in the absence of Rgd1. Whole genome sequencing of four independent lineages revealed that amplification of chromosome 7 and the right portion of chromosome 3 can restore resistance in strains lacking Rgd1, suggesting that Rgd1 may enable azole resistance by influencing the expression or activity of genes in these amplified regions. Consistent with this model, we found that overexpression of the transporter gene *NPR2*, located on the amplified right portion of chromosome 3, was sufficient to restore azole resistance in a strain lacking Rgd1. Thus, we establish novel circuitry important for antifungal drug resistance, and implicate a new facet of genomic plasticity in pathogen adaptation to drug-induced stress.

Our findings highlight evolutionary divergence of circuitry underpinning azole resistance. Although there are many examples of circuitry rewiring between *S*. *cereivisae* and *C*. *albicans* [56–59], there is conservation of most canonical drug resistance mechanisms, including alteration of the drug target Erg11, activation of efflux pumps, and signaling through key hubs of cellular stress responses [6, 8]. As poignant examples, the molecular chaperone Hsp90, and its client proteins calcineurin and Slt2/Mkc1 have all been implicated in azole resistance of diverse fungi [15, 16, 60]. In contrast, we observed a conserved impact on azole resistance in *C*. *albicans* for only two of the nine genes for which deletion abrogates *erg3*-mediated resistance in *S*. *cerevisiae*; one encoding the transcriptional regulator Upc2 and the other encoding the catalytic subunit of calcineurin Cna1. This suggests that although key hubs in the genetic network governing drug resistance are conserved, there is extensive divergence in circuitry controlling crucial cellular responses to drug-induced stress. This resonates with previous findings that the circuitry downstream of both calcineurin and Slt2/Mkc1 enabling membrane stress responses have been rewired between *S*. *cerevisiae* and *C*. *albicans* [14–16]. The genetic evidence for divergence in resistance circuitry is also complemented by chemical biology screens, which have revealed distinct sets of molecules that potentiate azole activity against different fungal species [61, 62]. Together, this emphasizes the necessity of studying drug resistance directly in fungal pathogens.

Our functional genomic screen in *C*. *albicans* defined a new role for the retromer component, Pep8, in azole resistance mediated via effects on cellular demand for the protein phophastase calcineurin. The stability of calcineurin depends on Hsp90, which interacts with the catalytic subunit under basal conditions, stabilizing calcineurin and keeping it poised for activation [63]. *PEP8* has recently been shown to interact genetically with *HSP90* in *C*. *albicans* [64], which may reflect that perturbation of Pep8 induces cellular stress. Consistent with this model, mutants defective in retrograde signaling, including *vps15Δ/vps15Δ*, *vps51Δ/vps51Δ* and *pep8Δ/pep8Δ* are hypersensitive to cell membrane stress [43]. Defects in endosomal trafficking have also been shown to modulate stress response signaling, with homozygous deletion of *VPS21*, a gene important for membrane trafficking through the late endosome/prevacuolar compartment, leading to increased intracellular calcium levels and enhanced expression of calcineurin effectors [65]. Strikingly, loss-of-function of *VPS21* leads to enhanced azole tolerance [65, 66], the opposite of what we observed upon loss of Pep8, highlighting complex functional relationships between regulators of endosomal transport and cell membrane stress responses.

The complexity of circuitry governing cellular responses to membrane stress is further emphasized by our discovery of Rgd1 as a novel effector of azole resistance that operates independent of established resistance determinants. Our results suggest that Rgd1 enables *erg3-*mediated azole resistance independent of its cognate GTPases Rho3 and Rho4, and independent of Pikα. By leveraging selection for genetic alterations that restore azole resistance in strains lacking Rgd1 coupled to whole genome sequencing, we discovered a new link between genomic plasticity and azole resistance. Exposure to fluconazole is known to promote aneuploidy formation in *C*. *albicans* due to alterations in cell cycle progression and the establishment of multinucleate trimera intermediates that are often resolved as aneuploid progeny [67]. The most well-characterized aneuploidy contributing to fluconazole resistance involves an isochromosome formation of the left arm of chromosome 5, which leads to azole resistance via amplification of genes encoding the drug target Erg11 and the efflux pump transcriptional regulator Tac1 [10–12]. Although we were unable to obtain isolates with restored azole resistance in an *erg3Δ/erg3Δ* *rgd1Δ/rgd1Δ* background, such isolates were readily obtained in the late clinical isolate CaCi-17 with both *RGD1* alleles deleted and all of the independent isolates shared a common aneuploidy signature with amplification of chromosome 7 and the right portion of chromosome 3. Such aneuploidies of the smaller chromosome have been associated with azole resistance [12, 68], including increased copy number of chromosomes 3 and 7, although the genes on these chromosomes important for enabling resistance remain largely enigmatic. Our analysis demonstrated that overexpression of the putative urea transporter gene *NPR2*, which resides on chromosome 3, can restore azole resistance in the absence of Rgd1. Although *C*. *albicans* aneuploidies often do not come at a fitness cost [12], we observed that the azole-resistant aneuploid isolates recovered in the clinical isolate background lacking Rgd1 had a fitness cost in the absence of drug and we were unable to recover resistant isolates in the background lacking both Erg3 and Rgd1. Thus, fitness constraints may limit the evolution of drug resistance in the absence of Rgd1, suggesting that it may be an attractive target for thwarting the emergence of drug resistance.

In this era of antimicrobial resistance, there is a pressing need to identify novel strategies to block the evolution of drug resistance and abrogate resistance once it has evolved. Defining the genes and genetic circuitry required for drug resistance provides a powerful approach for the rational design of combination therapies to achieve this goal [69]. Classic examples of this approach, include the molecular chaperone Hsp90, protein phosphatase calcineurin, and protein kinase Pkc1 [15, 16, 18]. The central challenge for targeting these cellular regulators for treatment of fungal infectious disease arises from their conservation and importance in the human host, necessitating the development of inhibitors that can selectively engage with the fungal counterpart [15, 70, 71]. Our discovery that Rgd1 and Pep8 regulate drug resistance expands the target space for antifungal drug development, and highlights that we are far from saturation given that screens with limited genomic coverage continue to yield novel resistance regulators. Progress with the development of more comprehensive genomic resources for fungal pathogens will enable elegant studies to elucidate the genetic interaction networks governing cellular stress response and drug resistance [26, 72, 73]. These genomic advances coupled with novel approaches to chemical synthesis and chemical biology screens will have a transformative impact on the discovery of molecules and mechanisms to evade drug resistance and treat life-threatening infectious disease [74–76].

## Materials & methods

### Strains and culture conditions

Strain stocks were maintained at -80°C in 25% glycerol. All strains were cultured in YPD (2% bactopeptone, 1% yeast extract, and 2% glucose) for liquid culture growth, or YPD with 2% agar for solid media growth at 30°C unless otherwise specified. Strains used in this study are listed in S3 Table. Cloning procedures were performed following standard protocols. Plasmids used in this study are listed in S4 Table. The absence of nonsynonymous mutations in plasmids was verified by sequencing. Primers used in this study are listed in S5 Table.

### Strain construction

#### CaLC2372

To generate a homozygous *UME6* deletion mutant in an *erg3Δ/erg3Δ* background, the NAT cassette was PCR amplified from pLC49 using primers oLC2398 and oLC2399 that contain homology to the *UME6* locus, and transformed into CaLC660. NAT resistant transformants were PCR tested for proper integration using oLC275 and oLC2400 as well as oLC274 and oLC2401. The *SAP2* promoter was induced to drive expression of the FLP recombinase to excise the NAT marker. NAT sensitive clones were transformed once more with the PCR amplified NAT cassette to delete the second *UME6* allele. NAT resistant transformants were PCR tested for proper integration using oLC275 and oLC2400 as well as oLC274 and oLC2401. To ensure the cassette deleted the intact allele rather than the previously deleted allele, the presence of the previously deleted allele was PCR tested using oLC2401 and oLC2400. The *SAP2* promoter was induced to drive expression of the FLP recombinase to excise the NAT marker cassette. The absence of a wild type allele was confirmed using oLC2400 and oLC2402.

#### CaLC2379

To generate a homozygous *UPC2* deletion mutant in an *erg3Δ/erg3Δ* background, the NAT cassette was PCR amplified from pLC49 using primers oLC2403 and oLC2404 that contain homology to the *UPC2* locus, and transformed into CaLC660. NAT resistant transformants were PCR tested for proper integration using oLC275 and oLC2405 as well as oLC274 and oLC2406. The *SAP2* promoter was induced to drive expression of the FLP recombinase to excise the NAT marker. NAT sensitive clones were transformed once more with the PCR amplified NAT cassette to delete the second *UPC2* allele. NAT resistant transformants were PCR tested for proper integration using oLC275 and oLC2405 as well as oLC274 and oLC2406. To ensure the cassette deleted the intact allele rather than the previously deleted allele, the presence of the previously deleted allele was PCR tested using oLC2405 and oLC2406. The absence of a wild type allele was confirmed with oLC2405 and oLC2407.

#### CaLC2438

To generate a homozygous *SST2* deletion mutant in an *erg3Δ/erg3Δ* background, the NAT cassette was PCR amplified from pLC49 using primers oLC2393 and oLC2394 that contain homology to the *SST2* locus, and transformed into CaLC660. NAT resistant transformants were PCR tested for proper integration using oLC275 and oLC2395 as well as oLC274 and oLC2396. To delete the second *SST2* allele, the Cd*HIS1* cassette was PCR amplified from pLC44 [77] using primers oLC2528 and oLC2529 that contain homology to the *SST2* locus. Transformants prototrophic for histidine were PCR tested for proper integration using oLC384 and oLC2395 as well as oLC385 and oLC2396. The absence of a wild type allele was confirmed using oLC2395 and oLC2397.

#### CaLC2439

To generate a homozygous *SIN4* deletion mutant in an *erg3Δ/erg3Δ* background, pLC704 was digested with KpnI and SacI to liberate the *SIN4* knock out cassette and was transformed into CaLC660. NAT resistant transformants were PCR tested for proper integration using oLC275 and oLC2422 as well as oLC274 and oLC2425. The *SAP2* promoter was induced to drive expression of the FLP recombinase to excise the NAT marker. NAT sensitive clones were transformed once more with the digested pLC704 construct to delete the second *SIN4* allele. NAT resistant transformants were PCR tested for proper integration using oLC275 and oLC2422 as well as oLC274 and oLC2425. To ensure the cassette deleted the intact allele rather than the previously deleted allele, the presence of the previously deleted allele was PCR tested using oLC2422 and oLC2425. The *SAP2* promoter was induced to drive expression of the FLP recombinase to excise the NAT marker. The absence of a wild type allele was confirmed with oLC2422 and oLC2387.

#### CaLC2559

To generate a homozygous *ERG3* deletion mutant in a *pho2Δ/pho2Δ* background, pLC361 was digested with KpnI and SacI to liberate the *ERG3* knock out cassette and was transformed into *pho2Δ/pho2Δ* Homann library deletion strain [78]. NAT resistant transformants were PCR tested for proper integration using oLC275 and oLC499 as well as oLC274 and oLC500. The *SAP2* promoter was induced to drive expression of the FLP recombinase to excise the NAT marker. NAT sensitive clones were transformed once more with pLC361 digested with KpnI and SacI to delete the remaining *ERG3* allele. NAT resistant transformants were PCR tested for proper integration using oLC275 and oLC499 as well as oLC274 and oLC500. To ensure the cassette deleted the intact allele rather than the previously deleted allele, the presence of the previously deleted allele was PCR tested using oLC499 and oLC500. The *SAP2* promoter was induced to drive expression of the FLP recombinase to excise the NAT marker. Absence of a wild type allele was confirmed using oLC499 and oLC166.

#### CaLC2760

To generate a heterozygous *SPT3* deletion mutant in an *erg3Δ/erg3Δ* background, the NAT cassette was PCR amplified from pLC49 using primers oLC2388 and oLC2389 that contain homology to the *SPT3* locus, and transformed into CaLC660. NAT resistant transformants were PCR tested for proper integration using oLC275 and oLC2390 as well as oLC274 and oLC2391. The *SAP2* promoter was induced to drive expression of the FLP recombinase to excise the NAT marker. NAT sensitive clones were transformed once more to promoter replace the remaining *SPT3* allele with a tetracycline repressible promoter. To do so, the NAT cassette from pLC605 was amplified using primers oLC2663 and oLC2664, which contain homology to the promoter region of *SPT3*. NAT resistant transformants were PCR tested for proper integration using oLC534 and oLC2390 as well as oLC300 and oLC2392. To ensure the cassette replaced the promoter upstream of the wild type allele rather than upstream of the previously deleted allele, the presence of the previously deleted allele was PCR tested using oLC2390 and oLC2391. The *SAP2* promoter was induced to drive expression of the FLP recombinase to excise the NAT marker cassette.

#### CaLC2761

To generate a heterozygous *orf19*.*3473* deletion mutant in an *erg3Δ/erg3Δ* background, the NAT cassette was PCR amplified from pLC49 using primers oLC2373 and oLC2374 that contain homology to the *orf19*.*3473* locus, and transformed into CaLC660. NAT resistant transformants were PCR tested for proper integration using oLC275 and oLC2375 as well as oLC274 and oLC2376. The *SAP2* promoter was induced to drive expression of the FLP recombinase to excise the NAT marker. NAT sensitive clones were transformed once more to promoter replace the remaining *orf19*.*3473* allele with a tetracycline repressible promoter. To do so, the NAT cassette from pLC605 was amplified using primers oLC2665 and oLC2666, which contain homology to the promoter region of *orf19*.*3473*. NAT resistant transformants were PCR tested for proper integration using oLC534 and oLC2375 as well as oLC300 and oLC2377. To ensure the cassette replaced the promoter upstream of the wild type allele rather than upstream of the previously deleted allele, the absence of a wild type promoter upstream of a wild type allele was confirmed with oLC2375 and oLC2377. The *SAP2* promoter was induced to drive expression of the FLP recombinase to excise the NAT marker cassette.

#### CaLC2998

To generate a homozygous *ERG3* deletion mutant in a *pbs2Δ/pbs2Δ* background, pLC361 was digested with KpnI and SacI to liberate the *ERG3* knock out cassette and was transformed into the *pbs2Δ/pbs2Δ* Noble library deletion strain [34]. NAT resistant transformants were PCR tested for proper integration using oLC275 and oLC499 as well as oLC274 and oLC500. The *SAP2* promoter was induced to drive expression of the FLP recombinase to excise the NAT marker. NAT sensitive clones were transformed once more with pLC361 digested with KpnI and SacI to delete the remaining *ERG3* allele. NAT resistant transformants were PCR tested for proper integration using oLC275 and oLC499 as well as oLC274 and oLC500. To ensure the cassette deleted the intact allele rather than the previously deleted allele, the presence of the previously deleted allele was PCR tested using oLC499 and oLC500. The *SAP2* promoter was induced to drive expression of the FLP recombinase to excise the NAT marker. Absence of a wild type allele was confirmed using oLC499 and oLC166.

#### CaLC2999

To generate a homozygous *ERG3* deletion mutant in a *rgd1Δ/rgd1Δ* background, pLC361 was digested with KpnI and SacI to liberate the *ERG3* knock out cassette and was transformed into the *rgd1Δ/rgd1Δ* Noble library deletion strain [34]. NAT resistant transformants were PCR tested for proper integration using oLC275 and oLC499 as well as oLC274 and oLC500. The *SAP2* promoter was induced to drive expression of the FLP recombinase to excise the NAT marker. NAT sensitive clones were transformed once more with pLC361 digested with KpnI and SacI to delete the remaining *ERG3* allele. NAT resistant transformants were PCR tested for proper integration using oLC275 and oLC499 as well as oLC274 and oLC500. To ensure the cassette deleted the intact allele rather than the previously deleted allele, the presence of the previously deleted allele was PCR tested using oLC499 and oLC500. The *SAP2* promoter was induced to drive expression of the FLP recombinase to excise the NAT marker. Absence of a wild type allele was confirmed using oLC499 and oLC166.

#### CaLC3474

To generate a homozygous *ERG3* deletion mutant in a *gzf3Δ/gzf3Δ* background, pLC361 was digested with KpnI and SacI to liberate the *ERG3* knock out cassette and was transformed into the *gzf3Δ/gzf3Δ* Noble library deletion strain [34]. NAT resistant transformants were PCR tested for proper integration using oLC275 and oLC499 as well as oLC274 and oLC500. The *SAP2* promoter was induced to drive expression of the FLP recombinase to excise the NAT marker. NAT sensitive clones were transformed once more with pLC361 digested with KpnI and SacI to delete the remaining *ERG3* allele. NAT resistant transformants were PCR tested for proper integration using oLC275 and oLC499 as well as oLC274 and oLC500. To ensure the cassette deleted the intact allele rather than the previously deleted allele, the presence of the previously deleted allele was PCR tested using oLC499 and oLC500. The *SAP2* promoter was induced to drive expression of FLP recombinase to excise the NAT marker. Absence of a wild type allele was confirmed using oLC499 and oLC166.

#### CaLC3598

To generate a homozygous *ERG3* deletion mutant in a *ssk2Δ/ssk2Δ* background, pLC361 was digested with KpnI and SacI to liberate the *ERG3* knock out cassette and was transformed into the *ssk2Δ/ssk2Δ* Noble library deletion strain [34]. NAT resistant transformants were PCR tested for proper integration using oLC275 and oLC499 as well as oLC274 and oLC500. The *SAP2* promoter was induced to drive expression of the FLP recombinase to excise the NAT marker. NAT sensitive clones were transformed once more with pLC361 digested with KpnI and SacI to delete the remaining *ERG3* allele. NAT resistant transformants were PCR tested for proper integration using oLC275 and oLC499 as well as oLC274 and oLC500. To ensure the cassette deleted the intact allele rather than the previously deleted allele, the presence of the previously deleted allele was PCR tested using oLC499 and oLC500. The *SAP2* promoter was induced to drive expression of the FLP recombinase to excise the NAT marker. Absence of a wild type allele was confirmed using oLC499 and oLC166.

#### CaLC3599

To generate a homozygous *ERG3* deletion mutant in an *apm1Δ/apm1Δ* background, pLC361 was digested with KpnI and SacI to liberate the *ERG3* knock out cassette and was transformed into the *apm1Δ/apm1Δ* Noble library deletion strain [34]. NAT resistant transformants were PCR tested for proper integration using oLC275 and oLC499 as well as oLC274 and oLC500. The *SAP2* promoter was induced to drive expression of the FLP recombinase to excise the NAT marker. NAT sensitive clones were transformed once more with pLC361 digested with KpnI and SacI to delete the remaining *ERG3* allele. NAT resistant transformants were PCR tested for proper integration using oLC275 and oLC499 as well as oLC274 and oLC500. To ensure the cassette deleted the intact allele rather than the previously deleted allele, the presence of the previously deleted allele was PCR tested using oLC499 and oLC500. The *SAP2* promoter was induced to drive expression of the FLP recombinase to excise the NAT marker. Absence of a wild type allele was confirmed using oLC499 and oLC166.

#### CaLC3601

To generate a homozygous *RGD1* deletion mutant in clinical isolate CaCi-17, the NAT cassette was PCR amplified from pLC49 using primers oLC3159 and oLC3160 that contain homology to the *RGD1* locus, and transformed into CaLC91. NAT resistant transformants were PCR tested for proper integration using oLC275 and oLC3161 as well as oLC274 and oLC3163. The *SAP2* promoter was induced to drive expression of the FLP recombinase to excise the NAT marker. NAT sensitive clones were transformed once more with the NAT cassette PCR amplified from pLC49 using primers oLC3159 and oLC3160 to replace the remaining *RGD1* allele. NAT resistant transformants were PCR tested for proper integration using oLC275 and oLC3161 as well as oLC274 and oLC3163. To ensure the cassette deleted the intact allele rather than the previously deleted allele, the presence of the previously deleted allele was PCR tested using oLC3161 and oLC3163. The *SAP2* promoter was induced to drive expression of the FLP recombinase to excise the NAT marker. Absence of a wild type allele was confirmed using oLC3161 and oLC3162.

#### CaLC3602

To generate a homozygous *RGD1* deletion mutant in clinical isolate CaCi-2, the NAT cassette was PCR amplified from pLC49 using primers oLC3159 and oLC3160 that contain homology to the *RGD1* locus, and transformed into CaLC79. NAT resistant transformants were PCR tested for proper integration using oLC275 and oLC3161 as well as oLC274 and oLC3163. The *SAP2* promoter was induced to drive expression of the FLP recombinase to excise the NAT marker. NAT sensitive clones were transformed once more with the NAT cassette PCR amplified from pLC49 using primers oLC3159 and oLC3160 to replace the remaining *RGD1* allele. NAT resistant transformants were PCR tested for proper integration using oLC275 and oLC3161 as well as oLC274 and oLC3163. To ensure the cassette deleted the intact allele rather than the previously deleted allele, the presence of the previously deleted allele was PCR tested using oLC3161 and oLC3163. The *SAP2* promoter was induced to drive expression of the FLP recombinase to excise the NAT marker. Absence of a wild type allele was confirmed using oLC3161 and oLC3162.

#### CaLC3664

To generate a homozygous *ERG3* deletion mutant in a *pep8Δ/pep8Δ* background, pLC361 was digested with KpnI and SacI to liberate the *ERG3* knock out cassette and was transformed into the *pep8Δ/pep8Δ* Noble library deletion strain [34]. NAT resistant transformants were PCR tested for proper integration using oLC275 and oLC499 as well as oLC274 and oLC500. The *SAP2* promoter was induced to drive expression of the FLP recombinase to excise the NAT marker. NAT sensitive clones were transformed once more with pLC361 digested with KpnI and SacI to delete the remaining *ERG3* allele. NAT resistant transformants were PCR tested for proper integration using oLC275 and oLC499 as well as oLC274 and oLC500. To ensure the cassette deleted the intact allele rather than the previously deleted allele, the presence of the previously deleted allele was PCR tested using oLC499 and oLC500. The *SAP2* promoter was induced to drive expression of the FLP recombinase to excise the NAT marker. Absence of a wild type allele was confirmed using oLC499 and oLC166.

#### CaLC3805

To generate a homozygous *RHO3* deletion mutant, the NAT cassette was PCR amplified from pLC49 using primers oLC3596 and oLC3597 that contain homology to the *RHO3* locus, and transformed into CaLC2999. NAT resistant transformants were PCR tested for proper integration using oLC275 and oLC3598 as well as oLC274 and oLC3599. The *SAP2* promoter was induced to drive expression of the FLP recombinase to excise the NAT marker. NAT sensitive clones were transformed once more with the NAT cassette PCR amplified from pLC49 using primers oLC3596 and oLC3597 to replace the remaining *RHO3* allele. NAT resistant transformants were PCR tested for proper integration using oLC275 and oLC3598 as well as oLC274 and oLC3599. To ensure the cassette deleted the intact allele rather than the previously deleted allele, the presence of the previously deleted allele was PCR tested using oLC3598 and oLC3599. The *SAP2* promoter was induced to drive expression of the FLP recombinase to excise the NAT marker. Absence of a wild type allele was confirmed using oLC3598 and oLC3600.

#### CaLC4089

To generate a homozygous *RHO4* deletion mutant, the NAT cassette was PCR amplified from pLC49 using primers oLC3601 and oLC3602 that contain homology to the *RHO4* locus, and transformed into CaLC2999. NAT resistant transformants were PCR tested for proper integration using oLC275 and oLC3603 as well as oLC274 and oLC3604. The *SAP2* promoter was induced to drive expression of the FLP recombinase to excise the NAT marker. NAT sensitive clones were transformed once more with the NAT cassette PCR amplified from pLC49 using primers oLC3601 and oLC3602 to replace the remaining *RHO4* allele. NAT resistant transformants were PCR tested for proper integration using oLC275 and oLC3603 as well as oLC274 and oLC3604. To ensure the cassette deleted the intact allele rather than the previously deleted allele, the presence of the previously deleted allele was PCR tested using oLC3603 and oLC3604. The *SAP2* promoter was induced to drive expression of the FLP recombinase to excise the NAT marker. Absence of a wild type allele was confirmed using oLC3603 and oLC3605.

#### CaLC4089

To generate a homozygous *RHO4* deletion mutant, the NAT cassette was PCR amplified from pLC49 using primers oLC3601 and oLC3602 that contain homology to the *RHO4* locus, and transformed into CaLC2302. NAT resistant transformants were PCR tested for proper integration using oLC275 and oLC3603 as well as oLC274 and oLC3604. The *SAP2* promoter was induced to drive expression of the FLP recombinase to excise the NAT marker. NAT sensitive clones were transformed once more with the NAT cassette PCR amplified from pLC49 using primers oLC3601 and oLC3602 to replace the remaining *RHO4* allele. NAT resistant transformants were PCR tested for proper integration using oLC275 and oLC3603 as well as oLC274 and oLC3604. To ensure the cassette deleted the intact allele rather than the previously deleted allele, the presence of the previously deleted allele was PCR tested using oLC3603 and oLC3604. The *SAP2* promoter was induced to drive expression of the FLP recombinase to excise the NAT marker. Absence of a wild type allele was confirmed using oLC3603 and oLC3605.

#### CaLC4144

To generate a homozygous *PEP8* deletion mutant in clinical isolate CaCi-2, the NAT cassette was PCR amplified from pLC49 using primers oLC3614 and oLC3615 that contain homology to the *PEP8* locus, and transformed into CaLC79. NAT resistant transformants were PCR tested for proper integration using oLC275 and oLC3616 as well as oLC274 and oLC3805. The *SAP2* promoter was induced to drive expression of the FLP recombinase to excise the NAT marker. NAT sensitive clones were transformed once more with the NAT cassette PCR amplified from pLC49 using primers oLC3614 and oLC3615 to replace the remaining *PEP8* allele. NAT resistant transformants were PCR tested for proper integration using oLC275 and oLC3616 as well as oLC274 and oLC3805. To ensure the cassette deleted the intact allele rather than the previously deleted allele, the presence of the previously deleted allele was PCR tested using oLC3616 and oLC3805. The *SAP2* promoter was induced to drive expression of the FLP recombinase to excise the NAT marker. Absence of a wild type allele was confirmed using oLC3455 and oLC3616.

#### CaLC4172

To generate a homozygous *PEP8* deletion mutant in clinical isolate CaCi-17, the NAT cassette was PCR amplified from pLC49 using primers oLC3614 and oLC3615 that contain homology to the *PEP8* locus, and transformed into CaLC91. NAT resistant transformants were PCR tested for proper integration using oLC275 and oLC3616 as well as oLC274 and oLC3805. The *SAP2* promoter was induced to drive expression of the FLP recombinase to excise the NAT marker. NAT sensitive clones were transformed once more with the NAT cassette PCR amplified from pLC49 using primers oLC3614 and oLC3615 to replace the remaining *PEP8* allele. NAT resistant transformants were PCR tested for proper integration using oLC275 and oLC3616 as well as oLC274 and oLC3805. To ensure the cassette deleted the intact allele rather than the previously deleted allele, the presence of the previously deleted allele was PCR tested using oLC3616 and oLC3805. The *SAP2* promoter was induced to drive expression of the FLP recombinase to excise the NAT marker. Absence of a wild type allele was confirmed using oLC3455 and oLC3616.

#### CaLC4235

To generate a homozygous *ERG3* deletion mutant in a *mrr2Δ/mrr2Δ* background, pLC361 was digested with KpnI and SacI to liberate the *ERG3* knock out cassette and was transformed into the *mrr2Δ/mrr2Δ* Noble library deletion strain (also referred to as *zcf342Δ/zcf342Δ*) [34]. NAT resistant transformants were PCR tested for proper integration using oLC275 and oLC499 as well as oLC274 and oLC500. The *SAP2* promoter was induced to drive expression of the FLP recombinase to excise the NAT marker. NAT sensitive clones were transformed once more with pLC361 digested with KpnI and SacI to delete the remaining *ERG3* allele. NAT resistant transformants were PCR tested for proper integration using oLC275 and oLC499 as well as oLC274 and oLC500. To ensure the cassette deleted the intact allele rather than the previously deleted allele, the presence of the previously deleted allele was PCR tested using oLC499 and oLC500. The *SAP2* promoter was induced to drive expression of FLP recombinase to excise the NAT marker. Absence of a wild type allele was confirmed using oLC499 and oLC166.

#### CaLC4339

To generate a homozygous *ERG3* deletion mutant in a *kic1Δ/kic1Δ* background, pLC361 was digested with KpnI and SacI to liberate the *ERG3* knock out cassette and was transformed into the *kic1Δ/kic1Δ* Noble library deletion strain [34]. NAT resistant transformants were PCR tested for proper integration using oLC275 and oLC499 as well as oLC274 and oLC500. The *SAP2* promoter was induced to drive expression of the FLP recombinase to excise the NAT marker. NAT sensitive clones were transformed once more with pLC361 digested with KpnI and SacI to delete the remaining *ERG3* allele. NAT resistant transformants were PCR tested for proper integration using oLC275 and oLC499 as well as oLC274 and oLC500. To ensure the cassette deleted the intact allele rather than the previously deleted allele, the presence of the previously deleted allele was PCR tested using oLC499 and oLC500. The *SAP2* promoter was induced to drive expression of FLP recombinase to excise the NAT marker. Absence of a wild type allele was confirmed using oLC499 and oLC166.

#### CaLC4349

A single and distinct colony of CaLC3601 was grown overnight in YPD. The next morning, cells were diluted to 2x10^9^ cells/mL, and 200 μl of diluted culture was plated on SD agar supplemented with arginine and 18 μg/mL of miconazole. Plates were incubated at 30°C for 5 days to allow for colony growth. Individual colonies were selected and elevated azole resistance was confirmed by performing a fluconazole MIC assay.

#### CaLC4353

A single and distinct colony of CaLC3601 was grown overnight in YPD. The next morning, cells were diluted to 2x10^9^ cells/mL, and 200 μl of diluted culture was plated on YPD agar with 18 μg/mL of miconazole. Plates were incubated at 30°C for 5 days to allow for colony growth. Individual colonies were selected and elevated azole resistance was confirmed by performing a fluconazole MIC assay.

#### CaLC4356

A single and distinct colony of CaLC3601 was grown overnight in YPD. The next morning, cells were diluted to 2x10^9^ cells/mL, and 200 μl of diluted culture was plated on YPD agar with 20 μg/mL of miconazole. Plates were incubated at 30°C for 5 days to allow for colony growth. Individual colonies were selected and elevated azole resistance was confirmed by performing a fluconazole MIC assay.

#### CaLC4358

A single and distinct colony of CaLC3601 was grown overnight in YPD. The next morning, cells were diluted to 2x10^9^ cells/mL, and 200 μl of diluted culture was plated on YPD agar with 20 μg/mL of miconazole. Plates were incubated at 30°C for 5 days to allow for colony growth. Individual colonies were selected and elevated azole resistance was confirmed by performing a fluconazole MIC assay.

#### CaLC4452

To generate a homozygous *ERG3* deletion mutant in a *rho3*Δ*/rho3*Δ** background, pLC361 was digested with KpnI and SacI to liberate the *ERG3* knock out cassette and was transformed into CaLC4861. NAT resistant transformants were PCR tested for proper integration using oLC275 and oLC499 as well as oLC274 and oLC500. The *SAP2* promoter was induced to drive expression of the FLP recombinase to excise the NAT marker. NAT sensitive clones were transformed once more with pLC361 digested with KpnI and SacI to delete the remaining *ERG3* allele. NAT resistant transformants were PCR tested for proper integration using oLC275 and oLC499 as well as oLC274 and oLC500. To ensure the cassette deleted the intact allele rather than the previously deleted allele, the presence of the previously deleted allele was PCR tested using oLC499 and oLC500. The *SAP2* promoter was induced to drive expression of the FLP recombinase to excise the NAT marker. Absence of a wild type allele was confirmed using oLC499 and oLC166.

#### CaLC4505

To generate a homozygous *ERG3* deletion mutant in a *rho4*Δ*/rho4*Δ** background, pLC361 was digested with KpnI and SacI to liberate the *ERG3* knock out cassette and was transformed into CaLC4141. NAT resistant transformants were PCR tested for proper integration using oLC275 and oLC499 as well as oLC274 and oLC500. The *SAP2* promoter was induced to drive expression of the FLP recombinase to excise the NAT marker. NAT sensitive clones were transformed once more with pLC361 digested with KpnI and SacI to delete the remaining *ERG3* allele. NAT resistant transformants were PCR tested for proper integration using oLC275 and oLC499 as well as oLC274 and oLC500. To ensure the cassette deleted the intact allele rather than the previously deleted allele, the presence of the previously deleted allele was PCR tested using oLC499 and oLC500. The *SAP2* promoter was induced to drive expression of the FLP recombinase to excise the NAT marker. Absence of a wild type allele was confirmed using oLC499 and oLC166.

#### CaLC4731

To generate a homozygous *PIK*α deletion mutant, the NAT cassette was PCR amplified from pLC49 using primers oLC4629 and oLC4630 that contain homology to the *PIK*α locus, and transformed into CaLC2302. NAT resistant transformants were PCR tested for proper integration using oLC275 and oLC4632 as well as oLC274 and oLC4633. The *SAP2* promoter was induced to drive expression of the FLP recombinase to excise the NAT marker. NAT sensitive clones were transformed once more with the NAT cassette PCR amplified from pLC49 using primers oLC4629 and oLC4630 to replace the remaining *PIK*α allele. NAT resistant transformants were PCR tested for proper integration using oLC275 and oLC4632 as well as oLC274 and oLC4633. To ensure the cassette deleted the intact allele rather than the previously deleted allele, the presence of the previously deleted allele was PCR tested using oLC4632 and oLC4633. The *SAP2* promoter was induced to drive expression of the FLP recombinase to excise the NAT marker. Absence of a wild type allele was confirmed using oLC4631 and oLC4633.

#### CaLC4788

To generate a homozygous *ERG3* deletion mutant in a *pik*α*Δ/pik*α*Δ* background, pLC361 was digested with KpnI and SacI to liberate the *ERG3* knock out cassette and was transformed into CaLC4731. NAT resistant transformants were PCR tested for proper integration using oLC275 and oLC499 as well as oLC274 and oLC500. The *SAP2* promoter was induced to drive expression of the FLP recombinase to excise the NAT marker. NAT sensitive clones were transformed once more with pLC361 digested with KpnI and SacI to delete the remaining *ERG3* allele. NAT resistant transformants were PCR tested for proper integration using oLC275 and oLC499 as well as oLC274 and oLC500. To ensure the cassette deleted the intact allele rather than the previously deleted allele, the presence of the previously deleted allele was PCR tested using oLC499 and oLC500. The *SAP2* promoter was induced to drive expression of the FLP recombinase to excise the NAT marker. Absence of a wild type allele was confirmed using oLC499 and oLC166.

#### CaLC4861

To generate a homozygous *RHO3* deletion mutant, the NAT cassette was PCR amplified from pLC49 using primers oLC3596 and oLC3597 that contain homology to the *RHO3* locus, and transformed into CaLC2302. NAT resistant transformants were PCR tested for proper integration using oLC275 and oLC3598 as well as oLC274 and oLC3599. The *SAP2* promoter was induced to drive expression of the FLP recombinase to excise the NAT marker. NAT sensitive clones were transformed once more with the NAT cassette PCR amplified from pLC49 using primers oLC3596 and oLC3597 to replace the remaining *RHO3* allele. NAT resistant transformants were PCR tested for proper integration using oLC275 and oLC3598 as well as oLC274 and oLC3599. To ensure the cassette deleted the intact allele rather than the previously deleted allele, the presence of the previously deleted allele was PCR tested using oLC3598 and oLC3599. The *SAP2* promoter was induced to drive expression of the FLP recombinase to excise the NAT marker. Absence of a wild type allele was confirmed using oLC3598 and oLC3600.

#### CaLC4863

To generate a homozygous *ERG3* deletion mutant in a strain with the regulatory subunit of calcineurin deleted (*CNA1*), pLC361 was digested with KpnI and SacI to liberate the *ERG3* knock out cassette and was transformed into CaLC2752. NAT resistant transformants were PCR tested for proper integration using oLC275 and oLC499 as well as oLC274 and oLC500. The *SAP2* promoter was induced to drive expression of the FLP recombinase to excise the NAT marker. NAT sensitive clones were transformed once more with pLC361 digested with KpnI and SacI to delete the remaining *ERG3* allele. NAT resistant transformants were PCR tested for proper integration using oLC275 and oLC499 as well as oLC274 and oLC500. To ensure the cassette deleted the intact allele rather than the previously deleted allele, the presence of the previously deleted allele was PCR tested using oLC499 and oLC500. The *SAP2* promoter was induced to drive expression of the FLP recombinase to excise the NAT marker. Absence of a wild type allele was confirmed using oLC499 and oLC166.

#### CaLC4864

To generate a homozygous *ERG3* deletion mutant, pLC361 was digested with KpnI and SacI to liberate the *ERG3* knock out cassette and was transformed into CaLC2751. NAT resistant transformants were PCR tested for proper integration using oLC275 and oLC3166 as well as oLC274 and oLC500. The *SAP2* promoter was induced to drive expression of the FLP recombinase to excise the NAT marker. NAT sensitive clones were transformed once more with pLC361 digested with KpnI and SacI to delete the remaining *ERG3* allele. NAT resistant transformants were PCR tested for proper integration using oLC275 and oLC3166 as well as oLC274 and oLC500. To ensure the cassette deleted the intact allele rather than the previously deleted allele, the presence of the previously deleted allele was PCR tested using oLC3166 and oLC500. The *SAP2* promoter was induced to drive expression of the FLP recombinase to excise the NAT marker. Absence of a wild type allele was confirmed using oLC3166 and oLC3167.

#### CaLC5281

To generate a strain with the promoter of one allele of *NPR2* replaced with a tetracycline repressible promoter in a CaLC3601 background, the TAR-tetO and NAT cassette was amplified from pLC605 using primers oLC6351/oLC6352, which contain homology to the promoter region of *NPR2*. NAT resistant transformants were PCR tested for proper integration using oLC4714 and oLC6354 as well as oLC534 and oLC6353. To determine if the cassette replaced the promoter upstream of the wild type one or both alleles, the absence or presence of a wild type promoter upstream of the wild type allele was confirmed with oLC6353 and oLC6354. The *SAP2* promoter was induced to drive expression of the FLP recombinase to excise the NAT marker cassette.

#### CaLC5283

To generate a strain with the promoter of one allele of *orf19*.*304* replaced with a tetracycline repressible promoter in a CaLC3601 background, the TAR-tetO and NAT cassette was amplified from pLC605 using primers oLC6357/oLC6358, which contain homology to the promoter region of *orf19*.*304*. NAT resistant transformants were PCR tested for proper integration using oLC4714 and oLC6360 as well as oLC534 and oLC6359. To determine if the cassette replaced the promoter upstream of the wild type one or both alleles, the absence or presence of a wild type promoter upstream of the wild type allele was confirmed with oLC6359 and oLC6360. The *SAP2* promoter was induced to drive expression of the FLP recombinase to excise the NAT marker cassette.

#### CaLC5313

To generate a strain with the promoter of one allele of *orf19*.*344* replaced with a tetracycline repressible promoter in a CaLC3601 background, the TAR-tetO and NAT cassette was amplified from pLC605 using primers oLC6363/oLC6364, which contain homology to the promoter region of *orf19*.*344*. NAT resistant transformants were PCR tested for proper integration using oLC4714 and oLC6366 as well as oLC534 and oLC6365. To determine if the cassette replaced the promoter upstream of the wild type one or both alleles, the absence or presence of a wild type promoter upstream of the wild type allele was confirmed with oLC6365 and oLC6366. The *SAP2* promoter was induced to drive expression of the FLP recombinase to excise the NAT marker cassette.

#### CaLC4349*-*A

A single and distinct colony of CaLC4349 was grown overnight in YPD. The next morning, cells were diluted and 200 μl of culture was plated on YPD agar. Plates were incubated at 30°C for 3 days to allow for individual colony growth. Individual colonies were selected and analyzed by whole genome sequencing to identify strains with resorted diploid levels of chromosome 7.

#### ScLC1660

To restore the wild type copy of *PHO2* in a *pho2*Δ** mutant, ScLC1601 was mated with ScLC9. The diploids were sporulated and tetrads were dissected. To ensure that wild type *PHO2* was restored, tetrads were replica plated onto YPD + G418 plates. G418 sensitive tetrads were PCR tested with oLC1261 in combination with oLC1548 to confirm a wild type *PHO2* allele was present.

#### ScLC1661

To restore the wild type copy of *SPT3* in a *spt3*Δ** mutant, ScLC1603 was mated with ScLC9. The diploids were sporulated and tetrads were dissected. To ensure that wild type *SPT3* was restored, tetrads were replica plated onto YPD + G418 plates. G418 sensitive tetrads were PCR tested with oLC1268 in combination with oLC1533 to confirm a wild type *SPT3* allele was present.

#### ScLC1662

To restore the wild type copy of *UPC2* in a *upc2*Δ** mutant, ScLC1604 was mated with ScLC9. The diploids were sporulated and tetrads were dissected. To ensure that wild type *UPC2* was restored, tetrads were replica plated onto YPD + G418 plates. G418 sensitive tetrads were PCR tested with oLC1269 in combination with oLC1534 to confirm a wild type *UPC2* allele was present.

#### ScLC1663

To restore the wild type copy of *UME6* in a *ume6*Δ** mutant, ScLC1605 was mated with ScLC9. The diploids were sporulated and tetrads were dissected. To ensure that wild type *UME6* was restored, tetrads were replica plated onto YPD + G418 plates. G418 sensitive tetrads were PCR tested with oLC1270 in combination with oLC1535 to confirm a wild type *UME6* allele was present.

#### ScLC1664

To restore the wild type copy of *SGF73* in a *sgf73*Δ** mutant, ScLC1606 was mated with ScLC9. The diploids were sporulated and tetrads were dissected. To ensure that wild type *SGF73* was restored, tetrads were replica plated onto YPD + G418 plates. G418 sensitive tetrads were PCR tested with oLC1276 in combination with oLC1536 to confirm a wild type *SGF73* allele was present.

#### ScLC1667

To restore the wild type copy of *CNB1* in a *cnb1*Δ** mutant, ScLC1608 was mated with ScLC9. The diploids were sporulated and tetrads were dissected. To ensure that wild type *CNB1* was restored, tetrads were replica plated onto YPD + G418 plates. G418 sensitive tetrads were PCR tested with oLC1510 in combination with oLC1538 to confirm a wild type *CNB1* allele was present.

#### ScLC1668

To restore the wild type copy of *SST2* in a *sst2*Δ** mutant, ScLC1610 was mated with ScLC9. The diploids were sporulated and tetrads were dissected. To ensure that wild type *SST2* was restored, tetrads were replica plated onto YPD + G418 plates. G418 sensitive tetrads were PCR tested with oLC1512 in combination with oLC1540 to confirm a wild type *SST2* allele was present.

#### ScLC1670

To restore the wild type copy of *SIN4* in a *sin4*Δ** mutant, ScLC1612 was mated with ScLC9. The diploids were sporulated and tetrads were dissected. To ensure that wild type *SIN4* was restored, tetrads were replica plated onto YPD + G418 plates. G418 sensitive tetrads were PCR tested with oLC1514 in combination with oLC1542 to confirm a wild type *SIN4* allele was present.

#### ScLC1691

To restore the wild type copy of *CSF1* in a *csf1*Δ** mutant, ScLC1609 was mated with ScLC9. The diploids were sporulated and tetrads were dissected. To ensure that wild type *CSF1* was restored, tetrads were replica plated onto YPD + G418 plates. G418 sensitive tetrads were PCR tested with oLC1511 in combination with oLC1549 to confirm a wild type *CSF1* allele was present.

#### pLC704

~500 base pairs of homology upstream of *SIN4* was PCR amplified from SC5314 genomic DNA with primers oLC2420 and oLC2421 and cloned into pLC49 at KpnI and ApaI. Presence of the insert was verified with oLC275 and oLC2420. ~500 base pairs of homology downstream of *SIN4* was PCR amplified from SC5314 genomic DNA with primers oLC2423 and oLC2424 and cloned into pLC49 at SacI and SacII. Presence of the insert was verified with oLC274 and oLC2424. The *SIN4* knock out cassette can be liberated by digestion with KpnI and SacI.

### *S*. *cerevisiae* SGA analysis

SGA analysis was conducted in biological duplicate as described in [21–24]. The fitness of double mutants was evaluated by automated measurement of colony size. The epsilon score measures the extent to which colony size of a double mutant deviates from the colony size expected from combining two mutations together. The data includes both negative (putative synthetic sick/lethal) and positive interactions (potential epistatic or suppression interactions) involving the gene(s) of interest. The magnitude of the score is indicative of the strength of interaction. Based on statistical analysis, we determined a default cutoff for our quantitative genetic interactions consisting of an SGA score ≤ -0.25 in YPD medium. This strict confidence threshold was applied based on previous genome-wide SGA studies [23–25], and based on analysis of biological duplicates. Further, strict normalization strategies were employed to obtain accurate fitness measurements, as described previously [25]. When comparing the genetic interaction profiles with other query strains, genetic profile similarity is based on Pearson correlation. To assess the double deletion mutants for azole sensitivity, *erg3*Δ** query strains were crossed with 4,309 non-essential deletion mutants to generate double deletion mutants via several automated selection steps. This *erg3* SGA library was grown overnight in YPD in 96-well plates at 30°C. The next morning 0.5 μL of culture was added to YPD without and with 400 ng/mL of miconazole. Strains were incubated 30°C for 48 hours at which point the absorbance was determined at 600 nm using a spectrophotometer (Molecular Devices) and corrected for background from the corresponding medium. Strains identified as hypersensitive to miconazole (growth defect >60%) were verified for their azole sensitivity by performing minimum inhibitory concentration assays with the azole fluconazole.

### *C*. *albicans* functional genomic screen

To identify regulators of *C*. *albicans* azole tolerance, a homozygous deletion mutant collection consisting of 1,152 mutant strains covering 674 genes or roughly ~11% of the *C*. *albicans* genome [34] was screened in the absence and presence of fluconazole. Each strain from the library was grown overnight in 200 μL of YPD in 96-well plates. The next morning, the cultures were diluted 1:100 by pinning 0.5 μL into 50 μL of YPD. The 1:100 dilution was then pinned (0.5 μL) into 200 μL of YPD or 200 μL of YPD with 0.6 μg/mL of fluconazole. The plates were incubated at 30°C for 48 hours, at which point OD_600_ was measured using a spectrophotometer (Molecular Devices) and corrected for background from the corresponding medium. Growth for each strain in the presence of fluconazole was normalized to growth in YPD alone. In total, 103 strains showed growth inhibition in fluconazole ≥ 75% compared to the no drug control. For genes for which multiple mutants were generated, the drug susceptibility phenotype of both mutants was confirmed. These were all tested for fluconazole susceptibility using a standard dose response minimum inhibitory concentration assay. The 13 strains that validated and showed the most robust phenotype were selected for follow-up analysis.

### Minimum inhibitory concentration (MIC) assay and growth kinetics assay

Antifungal susceptibility was measured in flat bottom, 96-well microtitre plates (Sarstedt # 83.3924) using a broth microdilution protocol described in [79]. In brief, minimum MIC assays were set up in 2-fold serial dilutions of fluconazole (Sequoia Research Products Ltd.), miconazole (Sigma-Aldrich), FK506 (A.G. Scientific, Inc.), geldanamycin (A.G. Scientific, Inc.), terbinafine (Sigma-Aldrich), SDS (Bioshop), amphotericin B (Sigma-Aldrich), or caspofungin (generously provided by Terry Roemer from Merck Research Laboratories) in a final volume of 200 μL per well. Where applicable, doxycycline (BD Biosciences #631311) was added to a final concentration of 1 μg/mL. All drug stocks were prepared in DMSO except for fluconazole, caspofungin, and SDS which were prepared in sterile ddH_2_O. Cell densities of overnight cultures were determined and dilutions were prepared such that ~10^3^ cells were inoculated into each well. Plates were incubated in the dark at 30°C for 24–48 hours as indicated, at which point the absorbance was determined at 600 nm using a spectrophotometer (Molecular Devices) and corrected for background from the corresponding medium. Growth was normalized to the no drug treatment well for the relevant strain, unless stated otherwise. Plotted are the average optical density values of technical duplicate measurements. Each strain was tested in duplicate in three biological replicates. MIC data was quantitatively displayed with color using the program Java TreeView 1.1.3 (http://jtreeview.sourceforge.net). For growth kinetics assays, cultures were grown overnight in YPD, diluted to an OD_600_ of 0.1 in 100 μL of YPD without or with fluconazole in 96-well plates, and grown at 30°C with continuous shaking (TECAN GENios). OD_595_ was measured every 15 minutes for 48 hours with XFluor4 software. All cultures were grown in technical triplicates. Growth curves were generated on at least two separate occasions. Data was plotted using GraphPad Prism.

### Western blot analysis

To assess the phosphorylation status of Mkc1 in response to fluconazole, overnight cultures of each strain were grown to saturation in YPD at 30°C. Cultures were then sub-cultured to an OD_600_ of 0.2 in 25 mL YPD, and allowed to grow for 3 hours at 30°C. Cultures were then split into two separate 10 mL cultures for treatment without or with fluconazole (8 μg/mL). Cultures were grown in the presence or absence of drug for 40 minutes at which point 1mL of cells was pelleted and washed in cold Tris buffered saline (TBS). Pellets were then re-suspended in 300 μL 0.1N sodium hydroxide, pelleted, and the supernatant removed. The pellet was re-suspended in 60 μL 1X sample buffer containing 0.35M Tris-HCl, 10% (w/w) SDS, 36% glycerol, 5% β-mercaptoethanol, and 0.012% bromophenol blue for SDS-PAGE. Samples were boiled at 95°C for 5 minutes and then separated on 8% SDS-PAGE gel. Proteins were transferred from the polyacrylamide to a PVDF membrane through electrotransfer (Bio-Rad laboratories Inc.). PVDF membranes were blocked with 5% BSA in TBS with 0.1% tween20 (TBS-T). To detect the phosphorylated form of Mkc1, an α-phospho-P44/42 MAPK (Thr202/Tyr204) antibody (Cell Signaling, 9102), was diluted 1:1500 and hybridized to the blot in the presence of 5% BSA in TBS-T. Actin was detected using an α-Act1 antibody (Santa Cruz Biotechnology, sc47778) at a 1:3333 dilution in 5% BSA in TBS-T.

### Quantitative RT-PCR

Overnight cultures of *C*. *albicans* grown in YPD at 30°C with shaking were diluted to an OD_600_ of 0.1 in a total volume of 10 mL YPD. Cells were grown for 2 hours, at which point fluconazole was added at a final concentration of 16 μg/mL. Cells were grown for an additional 2 hours. To monitor gene expression of evolved mutants, strains were subcultured to an OD_600_ of 0.1 and allowed to grow to mid-log phase. To prepare samples for RNA extraction, 10 mL of subculture was harvested by centrifugation at 1300g for 5 min. The pellet was washed with 2 mL cold ddH_2_O before being flash-frozen and stored at -80°C overnight. RNA was isolated using the QIAGEN RNeasy kit and cDNA was generated using the AffinityScript cDNA synthesis kit (Stratagene). qRT-PCR was carried out using the Fast SYBR Green Master Mix (Thermo Fisher Scientific) in 384-well plates with the following cycle conditions: 95°C for 10 min, repeat 95°C for 10 sec, 60°C for 30 sec for 40 cycles. The melt curve was completed with the following cycle conditions: 95°C for 10 sec and 65°C for 5 sec with an increase of 0.5°C per cycle up to 95°C. All reactions were done in triplicate and are representative of biological duplicates. Data were analyzed in the Bio-Rad CFX manager 3.1. Data was plotted and significance was assessed using GraphPad Prism.

### FM4-64 microscopy

For vacuole membrane staining with FM 4–64 (Thermo Fisher Scientific, # T3166), cells were grown overnight in YPD medium 30°C with agitation. The next morning, cells were diluted to an OD_600_ of 0.2 and allowed to grow for 3 hours. Cells were incubated with 8 μM FM 4–64 for 30 minutes at 30°C. Cells were washed and continuously pulsed with FM 4–64 for an additional 60 minutes with 8 μM FM 4–64. Cells were resuspended in SC media and visualized on a Zeiss Imager M1 upright microscope and AxioCam Mrm with AxioVision 4.7 software. For fluorescence microscopy, an X-cite series 120 light source with ET HQ tetramethylrhodamine isothiocyanate (TRITC)/DsRED filter set from Chroma Technology (Bellows Falls, VT) was used.

### Selection of azole-resistant mutants

For selection of azole resistance by a rapid, one-step regime, CaLC3601 (CaCi-17 *rgd1*Δ*/rgd1*Δ**) was grown overnight in YPD at 30°C. To establish overnight cultures, four independent colonies were selected to establish four independent lineages. Cell counts were performed with a hemacytometer, and ∼2 x 10^9^ cells were plated on SD agar or YPD agar supplemented with 18–20 μg/mL miconazole (Sigma-Aldrich). Plates were left to incubate for 5 days at 30°C in the dark before individual colonies were selected for drug susceptibility and whole genome sequencing analyses. To identify mutants that had lost the chromosome 7 aneuploidy, a single colony of CaLC4349 (CaCi-17 *rgd1*Δ*/rgd1*Δ** R1) was grown overnight in YPD and cultures were plated for single colonies on YPD agar in the absence of drug. Plates were left to incubate for 3 days at 30°C in the dark before individual colonies were selected for whole genome sequencing analysis.

### Genome sequencing analysis

Genomic DNA was isolated with phenol chloroform, as described previously [80]. Libraries were prepared using the NexteraXT DNA Sample Preparation Kit following the manufacturer’s instructions (Illumina). Libraries were purified with AMPure XP beads (Agencourt) and library concentration was quantified using a Bioanalyzer High Sensitivity DNA Chip (Agilent Technologies) and a Qubit High Sensitivity dsDNA fluorometric quantification kit (Life Technologies). DNA Libraries were sequenced using paired end 2x250 flow cells on an Illumina MiSeq (Creighton University). Copy number variation was visualized using Y_MAP_ [81].

### Accession numbers

All genome sequencing information has been deposited into the NCBI BioProject Accession number: PRJNA323475.

## Supporting information

S1 Table *ERG3* genetic interaction network in *S*. *cerevisiae*. (XLS)

S2 Table Growth of *S*. *cerevisiae* SGA mutants that displayed hypersensitivity to miconazole. (XLSX)

S3 Table Strains used in this study. (DOCX)

S4 Table Plasmids used in this study. (DOCX)

S5 Table Oligonucleotides used in this study. (XLSX)

S1 Fig Drug sensitivity profiles for *rgd1*Δ*/*Δ** and *pep8*Δ*/*Δ** mutants. **A)** Microbroth dilution minimum inhibitory concentration (MIC) assays of wild type, *rgd1*Δ*/*Δ** and *pep8*Δ*/*Δ** mutants in response to diverse stresses. MIC assay was performed as described in Fig 1. Growth was measured after 24 hours. **B)** Histogram plots highlighting variation in OD_600_ values between technical duplicates for MIC plots shown in Fig 2D. Strains were grown in the absence or presence of 2 μg/mL of fluconazole (left plot) or 64 μg/mL (right plot). Optical densities were averaged for duplicate measurements and error bars represent standard deviation of duplicate measurements. MIC was performed in biological triplicate with similar results observed.
(TIF)

S2 Fig Deletion of *PEP8* results in fragmented vacuolar morphology in *C*. *albicans* azole-resistant backgrounds. **A)** Strains of *C*. *albicans* were grown to log phase prior to staining with membrane dye FM4-64. Images were captured using differential interference contrast (DIC) microscopy and fluorescence microscopy with a TRITC/DsRED filter set on a Zeiss Axio Observer.Z1 (Carl Zeiss) using 100x magnification. Scale bar represents 10 μm. **B)** Strains of *C*. *albicans* were grown to log-phase prior to staining with membrane dye FM4-64. Images were captured using differential interference contrast (DIC) microscopy and fluorescence microscopy with a TRITC/DsRED filter set on a Zeiss Axio Observer.Z1 (Carl Zeiss) using 100x magnification. Scale bar represents 10 μm.
(TIF)

S3 Fig Characterizing the role of Pep8 in orchestrating *erg3-*mediated resistance. **A)** Hyperactive calcineurin does not abrogate *erg3-*mediated azole resistance. Resistance mediated by loss of *ERG3* is maintained in a strain harbouring a hyperactive calcineurin allele (*CNA1-Tr*). MIC was performed as described in Fig 1. Growth was measured after 24 hours. **B)** Histogram plots highlighting variation in OD_600_ values between technical duplicates for MIC plots shown in Fig 3C. Strains were grown in the absence or presence of FK-506 (0.78 μM) or Geldanamycin (0.78 μM). Optical densities were averaged for duplicate measurements and error bars represent standard deviation of duplicate measurements. MIC was performed in biological triplicate with similar results observed.
(TIF)

S4 Fig Loss of *RGD1* enables azole resistance independent of known resistance mechanisms. **A)** Deletion of *RGD1* does not reduce expression of the azole target gene *ERG11*, nor the expression of efflux transporters *CDR1*, *CDR2*, or *MDR1*. Strains were grown in in YPD (-) or YPD with 16 μg/mL Fluconazole. Transcript levels were monitored by qRT-PCR and normalized to *GPD1* or *ACT1* as indicated. Error bars represent standard error of the mean for triplicate samples. **B)** Deletion of *ERG3* leads to activation of the cell wall integrity signaling under basal conditions, but deletion of *RGD1* does not block activation of cell wall integrity under basal conditions or in response to fluconazole (FL). Strains were left untreated or treated with 8 μg/mL FL for 40 minutes, as indicated. Phosphorylated Mkc1 (P-Mkc1) was monitored by Western blot and detected with an α-p44/42 antibody. Actin was detected with an α-β-actin antibody as a loading control. **C)** Deletion of *RGD1* does not reduce expression of calcineurin-dependent transcript, *UTR2*. Transcript levels were monitored by qRT-PCR and normalized to *GPD1*. Error bars represent standard error of the mean for triplicate samples. **D)** Deletion of putative *C*. *albicans RGD1* physical interactors, identified based on reports in *S*. *cerevisiae*, does not abrogate *erg3-*mediated resistance. MIC assay was performed as described in Fig 1. Growth was measured after 24 hours.
(TIF)

S5 Fig Transcript levels of candidate resistance determinants in aneuploid genomic regions with enhanced copy number, and geldanamycin susceptibility of *rgd1* azole-resistant mutants. **A)** Azole-resistant isolates (R1-R4) have increased expression of *HSP90*, *orf19*.*304*, and *orf19*.*344*, *FLU1*, *NPR2*, *HGT12*, and *HGT13* relative to the evolved parent. Transcript levels were monitored by qRT-PCR and normalized to *GPD1* or *ACT1* as indicated. Error bars represent standard error of the mean for triplicate samples. Expression levels of evolved strains were compared to the parental strain using a one-way ANOVA with Bonferroni post-test. Asterisk indicates significant difference in transcript level relative to the parental strain (* *P<*0.05). **B)** Spontaneous mutants show enhanced resistance to the Hsp90 inhibitor geldanamycin (GdA). Strains were grown in YPD medium in the absence (-) and presence of 25 μM GdA. Growth was measured by absorbance at 600 nm after 48 hours at 30°C and normalized to growth in the absence of inhibitor. Optical densities were averaged for duplicate measurements and error bars represent standard deviation of duplicate measurements.
(TIF)

S6 Fig Azole susceptibility profiles of *C*. *albicans* mutants correlates with intracellular rhodamine 6G accumulation. Deletion of *RGD1* increases rhodamine-6G accumulation in a *C*. *albicans* clinical isolate (CaCi-17), which correlates with enhanced sensitivity to azoles. Selection of four azole-resistant CaCi-17 *rgd1*Δ*/rgd1*Δ** lineages (R1-R4) results in decreased accumulation of rhodamine 6G. Scale bar represents 10 μm. Assay was performed in biological duplicates.
(TIF)
