# Supplementary material for: Global analysis of genetic circuitry and adaptive mechanisms enabling resistance to the azole antifungal drugs
Source: PLoS Genet. 2018 Apr 27;14(4):e1007319. doi: 10.1371/journal.pgen.1007319 (PMC5922528; doi:10.1371/journal.pgen.1007319)
Supplement: S3 Table — (DOCX) [file pgen.1007319.s003.docx]

**S3 Table: Strains used in this study.**

| Strain ID | Strain Name | Genotype | Source |
| --- | --- | --- | --- |
| **CaLC79** | Early C.I. (CaCi-2) | Clinical Isolate | [1] |
| **CaLC91** | Late C.I. (CaCi-17) | Clinical Isolate | [1] |
| **CaLC239** | SN95  Wildtype | *arg4/arg4 his1/his1 URA3/ ura3::imm434 IRO1/iro1 ::imm434* | [2] |
| **CaLC660** | *erg3Δ/erg3Δ* | *arg4*/*arg4* *his1*/*his1* *URA3*/ *ura3*::*imm434* *IRO1*/*iro1*::*imm434* *CaTAR::HIS3* *erg3*::FRT/*erg3*::FRT | [3] |
| **CaLC1458** | *erg3Δ/erg3Δ cna1Δ/cna1Δ* | *arg4/ arg4 his1/his1 URA3/ ura3::imm434 IRO1/iro1::imm434 CaTAR::HIS3 erg3::FRT/erg3::FRT cna1::FRT/cna1::FRT* | [4] |
| **CaLC2302** | SN250 Wildtype | *his1/his1 arg4/arg4 URA3/ ura3::imm434, IRO1/iro1::imm434 leu2::C.dubliniensis HIS1 /leu2 ::C.maltosa LEU2* | [5] |
| **CaLC2372** | *erg3Δ/erg3Δ ume6Δ/ume6Δ* | As CaLC660, *ume6::FRT/ume6::NAT* | This study |
| **CaLC2379** | *erg3Δ/erg3Δ upc2Δ/upc2Δ* | As CaLC660, *upc2::FRT/upc2::NAT* | This study |
| **CaLC2438** | *erg3Δ/erg3Δ sst2Δ/sst2Δ* | As CaLC660, *sst2::NAT/sst2::CdHIS1* | This study |
| **CaLC2439** | *erg3Δ/erg3Δ sin4Δ/sin4Δ* | As CaLC660, *sin4::FRT/sin4::FRT* | This study |
| **CaLC2559** | *erg3Δ/erg3Δ pho2Δ/pho2Δ* | *arg4/arg4 leu2/leu2 his1/his1 URA3/ ura3::imm434, IRO1/iro1::imm434 pho2::HIS2/pho2::LEU2 erg3::FRT/erg::FRT* | This study |
| **CaLC2751** | *Ca-cna1/CNA1* | *cnaD::hisG/cnaD::hisG LEU2::CNA::URA3* | [6] |
| **CaLC2752** | *Ca-cna1/CNAtr* | *cnaD::hisG/cnaD::hisG LEU2::CNAtr::URA3* | [6] |
| **CaLC2760** | *erg3Δ/erg3Δ tetO-SPT3/spt3Δ* | As CaLC660, *FRT-tetO-SPT3/spt3::FRT* | This study |
| **CaLC2761** | *erg3Δ/erg3Δ tetO-orf19.3473 /orf19.3473Δ* | As CaLC660, *FRT-tetO-orf19.3473/ orf19.3473::FRT* | This study |
| **CaLC2998** | *erg3Δ/erg3Δ*  *pbs2Δ/pbs2Δ* | *his1/his1 leu2/leu2 arg4/arg4 URA3/ura3:: imm434 IRO1/iro1::imm434 pbs2:: C.dubliniensis HIS1/pbs2::C.maltosa LEU2 erg3::*FRT*/erg3::*FRT | This study |
| **CaLC2999** | *erg3Δ/erg3Δ rgd1Δ/rgd1Δ* | *his1/his1 leu2/leu2 arg4/arg4 URA3/ura3:: imm434 IRO1/iro1::imm434 rgd1:: C.dubliniensis HIS1/rgd1::C.maltosa LEU2 erg3::*FRT*/erg3::*FRT | This study |
| **CaLC3403** | *orf19.2378Δ/orf19.2378Δ* | *his1/his1 leu2/leu2 arg4/arg4 URA3/ ura3::imm434 IRO1/iro1::imm434, orf19.2378::C.dubliniensis HIS1/orf19.2378::C.maltosa LEU2* | [5] |
| **CaLC3405** | *kic1Δ/kic1Δ* | *his1/his1 leu2/leu2 arg4/arg4 URA3/ ura3::imm434 IRO1/iro1::imm434 kic1::C.dubliniensis HIS1/kic1:: C.maltosa LEU2* | [5] |
| **CaLC3407** | *rcy1Δ/rcy1Δ* | *his1/his1 leu2/leu2 arg4/arg4 URA3/ ura3::imm434 IRO1/iro1::imm434 rcy1::C.dubliniensis HIS1/rcy1:: C.maltosa LEU2* | [5] |
| **CaLC3409** | *mrr2Δ/mrr2Δ* | *his1/his1 leu2/leu2 arg4/arg4 URA3/ ura3::imm434 IRO1/iro1::imm434 mrr2::C.dubliniensis HIS1/mrr2:: C.maltosa LEU2* | [5] |
| **CaLC3413** | *pep8Δ/pep8Δ* | *his1/his1 leu2/leu2 arg4/arg4 URA3/ ura3::imm434 IRO1/iro1::imm434 pep8::C.dubliniensis HIS1/pep8:: C.maltosa LEU2* | [5] |
| **CaLC3415** | *gzf3Δ/gzf3Δ* | *his1/his1 leu2/leu2 arg4/arg4 URA3/ ura3::imm434 IRO1/iro1::imm434 gzf3::C.dubliniensis HIS1/gzf3:: C.maltosa LEU2* | [5] |
| **CaLC3417** | *pbs2Δ/pbs2Δ* | *his1/his1 leu2/leu2 arg4/arg4 URA3 /ura3::imm434 IRO1/iro1::imm434 pbs2::C.dubliniensis HIS1/pbs2:: C.maltosa LEU2* | [5] |
| **CaLC3419** | *rgd1Δ/rgd1Δ* | *his1/his1 leu2/leu2 arg4/arg4 URA3 /ura3::imm434 IRO1/iro1::imm434 rgd1::C.dubliniensis HIS1/rgd1:: C.maltosa LEU2* | [5] |
| **CaLC3421** | *stt4Δ/stt4Δ* | *his1/his1 leu2/leu2 arg4/arg4 URA3 /ura3::imm434 IRO1/iro1::imm434 stt4::C.dubliniensis HIS1/stt4:: C.maltosa LEU2* | [5] |
| **CaLC3423** | *apm1Δ/apm1Δ* | *his1/his1 leu2/leu2 arg4/arg4 URA3/ ura3::imm434 IRO1/iro1::imm434 apm1::C.dubliniensis HIS1/apm1:: C.maltosa LEU2* | [5] |
| **CaLC3425** | *erg5Δ/erg5Δ* | *his1/his1 leu2/leu2 arg4/arg4 URA3/ ura3::imm434 IRO1/iro1::imm434 erg5::C.dubliniensis HIS1/erg5:: C.maltosa LEU2* | [5] |
| **CaLC3427** | *ssk2Δ/ssk2Δ* | *his1/his1 leu2/leu2 arg4/arg4 URA3 /ura3::imm434 IRO1/iro1::imm434 ssk2::C.dubliniensis HIS1/ssk2:: C.maltosa LEU2* | [5] |
| **CaLC3474** | *erg3Δ/erg3Δ*  *gzf3Δ/gzf3Δ* | As CaLC3415, *erg3::*FRT*/erg3∷*FRT | This study |
| **CaLC3598** | *erg3Δ/erg3Δ*  *ssk2Δ/ssk2Δ* | As CaLC3427, *erg3::*FRT*/erg3∷*FRT | This study |
| **CaLC3599** | *erg3Δ/erg3Δ*  *apm1Δ/apm1Δ* | As CaLC3423*, erg3::*FRT*/erg3∷*FRT | This study |
| **CaLC3601** | Late C.I.  *rgd1Δ/Δ* | As CaLC91, *rgd1*::FRT/*rgd1*∷FRT | This study |
| **CaLC3602** | Early C.I. *rgd1Δ/Δ* | As CaLC79, *rgd1*::FRT/*rgd1*∷FRT | This study |
| **CaLC3664** | *erg3Δ/erg3Δ pep8Δ/pep8Δ* | As CaLC3413, *erg3::*FRT*/erg3∷*FRT | This study |
| **CaLC3805** | *erg3Δ/erg3Δ rgd1Δ/rgd1Δ rho3Δ/rho3Δ* | As CaLC2999, *rho3∷*FRT*/rho3∷*NAT | This study |
| **CaLC4089** | *erg3Δ/erg3Δ rgd1Δ/rgd1Δ*  *rho4Δ/rho4Δ* | As CaLC2999*, rho4∷*FRT*/rho4∷*Nat | This study |
| **CaLC4141** | *rho4Δ/rho4Δ* | As CaLC2302, *rho4∷*FRT*/rho4∷*FRT | This study |
| **CaLC4144** | Early C.I. *pep8Δ/pep8Δ* | As CaLC79, *pep8*::FRT/*pep8*∷FRT | This study |
| **CaLC4172** | Late C.I. *pep8Δ/pep8Δ* | As CaLC91, *pep8*::FRT/*pep8*∷FRT | This study |
| **CaLC4235** | *erg3Δ/erg3Δ mrr2Δ/mrr2Δ* | *his1/his1 leu2/leu2 arg4/arg4 URA3/ ura3::imm434 IRO1/iro1::imm434 mrr2::C.dubliniensis HIS1/mrr2:: C.maltosa LEU2 erg3∷*FRT*/erg3::*FRT | This study |
| **CaLC4339** | *erg3Δ/erg3Δ*  *kic1Δ/kic1Δ* | *his1/his1 leu2/leu2 arg4/arg4 URA3/ ura3::imm434 IRO1/iro1::imm434 kic1::C.dubliniensis HIS1/kic1:: C.maltosa LEU2 erg3∷*FRT*/ erg3Δ∷*FRT | This study |
| **CaLC4349** | Resistant Isolate #1 | As CaLC3601, azole resistant | This study |
| **CaLC4353** | Resistant Isolate #2 | As CaLC3601, azole resistant | This study |
| **CaLC4356** | Resistant Isolate #3 | As CaLC3601, azole resistant | This study |
| **CaLC4358** | Resistant Isolate #4 | As CaLC3601, azole resistant | This study |
| **CaLC4452** | *erg3Δ/erg3Δ*  *rho3Δ/rho3Δ* | *his1/his1 leu2/leu2 arg4/arg4 URA3/ ura3::imm434 IRO1/iro1::imm434 rho3::C.dubliniensis HIS1/rho3:: C.maltosa LEU2 erg3::FRT/erg3∷FRT* | This study |
| **CaLC4505** | *erg3Δ/erg3Δ rho4Δ/rho4Δ* | As CaLC2302, *rho4∷*FRT*/rho4∷*FRT *erg3∷FRT/erg3∷FRT* | This study |
| **CaLC4731** | *pikαΔ/pikαΔ* | As CaLC2302, *pikα∷FRT/ pikα∷FRT* | This study |
| **CaLC4788** | *pikαΔ/pikαΔ erg3Δ/erg3Δ* | As CaLC4731, *erg3∷FRT/erg3∷FRT* | This study |
| **CaLC4861** | *rho3Δ/rho3Δ* | As CaLC2302,  *rho3∷FRT/rho3∷FRT* | This study |
| **CaLC4863** | *Ca-cna1/CNATr erg3Δ/erg3Δ* | As CaLC2752, *erg3∷FRT/erg3∷NAT* | This study |
| **CaLC4864** | *Ca-cna1/CNA1 erg3Δ/erg3Δ* | As CaLC2751, *erg3∷FRT/erg3∷FRT* | This study |
| **CaLC5281** | Late C.I. *rgd1Δ/Δ*  *tetO-NPR2/NPR2* | As CaLC3601, *FRT-tetO-NPR2/NPR2* | This study |
| **CaLC5283** | Late C.I. *rgd1Δ/Δ*  *tetO-orf19.304/orf19.304* | As CaLC3601, *FRT-tetO-orf19.304/orf19.304* | This study |
| **CaLC5313** | Late C.I. *rgd1Δ/Δ*  *tetO-orf19.344/orf19.344* | As CaLC3601, *FRT-tetO-orf19.344/orf19.344* | This study |
| **CaLC4349-A** | Resistant Isolate #1, diploid chromosome 7 | As CaLC4349, azole resistant, restored diploid chromosome 7 | This study |
| **ScLC10** | *erg3Δ* | As ScLC151, *erg3∷kanR* | [7] |
| **ScLC151** | BY4741 | *his3-1 leu2-O met15-O ura3-O* | [8] |
| **ScLC1601** | *erg3Δ pho2Δ* | As ScLC151, *erg3::natR pho2::kanR can1 ::MFA1pr-HIS3 lyp1* | Boone Lab via SGA |
| **ScLC1603** | *erg3Δ spt3Δ* | As ScLC151, *erg3::natR spt3::kanR can1 ::MFA1pr-HIS3 lyp1* | Boone Lab via SGA |
| **ScLC1604** | *erg3Δ upc2Δ* | As ScLC151, *erg3::natR upc2::kanR can1 ::MFA1pr-HIS3 lyp1* | Boone Lab via SGA |
| **ScLC1605** | *erg3Δ ume6Δ* | As ScLC151, *erg3::natR ume6::kanR can1 ::MFA1pr-HIS3 lyp1* | Boone Lab via SGA |
| **ScLC1606** | *erg3Δ sgf73Δ* | As ScLC151, *erg3 ::natR sgf73::kanR can1 ::MFA1pr-HIS3 lyp1* | Boone Lab via SGA |
| **ScLC1608** | *erg3Δ cnb1Δ* | As ScLC151, *erg3::natR cnb1::kanR can1 ::MFA1pr-HIS3 lyp1* | Boone Lab via SGA |
| **ScLC1609** | *erg3Δ csf1Δ* | As ScLC151, *erg3 ::natR csf1::kanR can1 ::MFA1pr-HIS3 lyp1* | Boone Lab via SGA |
| **ScLC1610** | *erg3Δ sst2Δ* | As ScLC151, *erg3::natR sst2::kanR can1::MFA1pr-HIS3 lyp1* | Boone Lab via SGA |
| **ScLC1612** | *erg3Δ sin4Δ* | As ScLC151, *erg3::natR sin4::kanR can1 ::MFA1pr-HIS3 lyp1* | Boone Lab via SGA |
| **ScLC1660** | *erg3Δ PHO2* | As ScLC151, *erg3::natR can1 ::MFA1pr-HIS3 lyp1* | This study |
| **ScLC1661** | *erg3Δ SPT3* | As ScLC151, *erg3::natR can1 ::MFA1pr-HIS3 lyp1* | This study |
| **ScLC1662** | *erg3Δ UPC2* | As ScLC151, *erg3::natR can1 ::MFA1pr-HIS3 lyp1* | This study |
| **ScLC1663** | *erg3Δ UME6* | As ScLC151, *erg3::natR can1 ::MFA1pr-HIS3 lyp1* | This study |
| **ScLC1664** | *erg3Δ SGF73* | As ScLC151, *erg3::natR can1 ::MFA1pr-HIS3 lyp1* | This study |
| **ScLC1667** | *erg3Δ CNB1* | As ScLC151, *erg3::natR can1 ::MFA1pr-HIS3 lyp1* | This study |
| **ScLC1668** | *erg3Δ SST2* | As ScLC151, *erg3::natR can1 ::MFA1pr-HIS3 lyp1* | This study |
| **ScLC1670** | *erg3Δ SIN4* | As ScLC151, *erg3::natR can1 ::MFA1pr-HIS3 lyp1* | This study |
| **ScLC1691** | *erg3Δ CSF1* | As ScLC151, *erg3::natR can1 ::MFA1pr-HIS3 lyp1* | This study |

**S3 Table References**

1. White TC. Increased mRNA levels of *ERG16, CDR*, and *MDR1* correlate with increases in azole resistance in *Candida albicans* isolates from a patient infected with human immunodeficiency virus. Antimicrob Agents Chemother. 1997;41(7):1482-7. PubMed PMID: 9210670.

2. Noble SM, Johnson AD. Strains and strategies for large-scale gene deletion studies of the diploid human fungal pathogen *Candida albicans.* Eukaryot Cell. 2005;4(2):298-309. PubMed PMID: 15701792.

3. Robbins N, Collins C, Morhayim J, Cowen LE. Metabolic control of antifungal drug resistance. Fungal Genet Biol. 2010;47(2):81-93. doi: 10.1016/j.fgb.2009.07.004. PubMed PMID: 19595784.

4. Hill JA, O'Meara TR, Cowen LE. Fitness Trade-Offs Associated with the evolution of resistance to antifungal drug combinations. Cell Rep. 2015; pii: S2211-1247(15)00010-8.

doi: 10.1016/j.celrep.2015.01.009. PubMed PMID: 25660029.

5. Noble SM, French S, Kohn LA, Chen V, Johnson AD. Systematic screens of a *Candida albicans* homozygous deletion library decouple morphogenetic switching and pathogenicity. Nat Genet. 2010;42(7):590-8. Epub 2010/06/15. doi: 10.1038/ng.605. PubMed PMID: 20543849; PubMed Central PMCID: PMC2893244.

6. Sanglard D, Ischer F, Marchetti O, Entenza J, Bille J. Calcineurin A of *Candida albicans:* involvement in antifungal tolerance, cell morphogenesis and virulence. Mol Microbiol. 2003;48(4):959-76. PubMed PMID: 12753189.

7. Cowen LE, Lindquist S. Hsp90 potentiates the rapid evolution of new traits: drug resistance in diverse fungi. Science. 2005;309(5744):2185-9. PubMed PMID: 16195452.

8. Giaever G, Chu AM, Ni L, Connelly C, Riles L, Veronneau S, et al. Functional profiling of the *Saccharomyces cerevisiae* genome. Nature. 2002;418(6896):387-91. PubMed PMID: 12140549.
