# Supplementary material for: Global analysis of genetic circuitry and adaptive mechanisms enabling resistance to the azole antifungal drugs
Source: PLoS Genet. 2018 Apr 27;14(4):e1007319. doi: 10.1371/journal.pgen.1007319 (PMC5922528; doi:10.1371/journal.pgen.1007319)
Supplement: S4 Table — (DOCX) [file pgen.1007319.s004.docx]

**S4 Table: Plasmids used in this study.**

| Name | Description | Reference |
| --- | --- | --- |
| pLC44 | pSN52  *C. dubliniensis HIS1* knock-out vector | [1] |
| pLC49 | p863  *FLP-CaNAT,* ampR | [2] |
| pLC361 | *CaERG3-K/O, NAT, amp^R^ (pLC49)* | [3] |
| pLC605 | *CaTAr-FLP-CaNAT, ampR* | [4] |
| pLC704 | *CaSIN4-K/O, NAT, amp^R^ (pLC49)* | This study |

**S4 Table References**

1. Noble SM, Johnson AD. Strains and strategies for large-scale gene deletion studies of the diploid human fungal pathogen *Candida albicans.* Eukaryot Cell. 2005;4(2):298-309. PubMed PMID: 15701792.

2. Shen J, Guo W, Kohler JR. Ca*NAT1*, a heterologous dominant selectable marker for transformation of *Candida albicans* and other pathogenic *Candida* species. Infect Immun. 2005;73(2):1239-42. PubMed PMID: 15664973.

3. Robbins N, Collins C, Morhayim J, Cowen LE. Metabolic control of antifungal drug resistance. Fungal Genet Biol. 2010;47(2):81-93. doi: 10.1016/j.fgb.2009.07.004. PubMed PMID: 19595784.

4. Leach MD, Cowen LE. Membrane fluidity and temperature sensing are coupled via circuitry comprised of Ole1, Rsp5, and Hsf1 in *Candida albicans*. Eukaryot Cell. 2014;13(8):1077-84. doi: 10.1128/EC.00138-14. PubMed PMID: 24951438; PubMed Central PMCID: PMCPMC4135801.
