# Supplementary figures and images for: Global analysis of genetic circuitry and adaptive mechanisms enabling resistance to the azole antifungal drugs
Source: PLoS Genet. 2018 Apr 27;14(4):e1007319. doi: 10.1371/journal.pgen.1007319 (PMC5922528; doi:10.1371/journal.pgen.1007319)

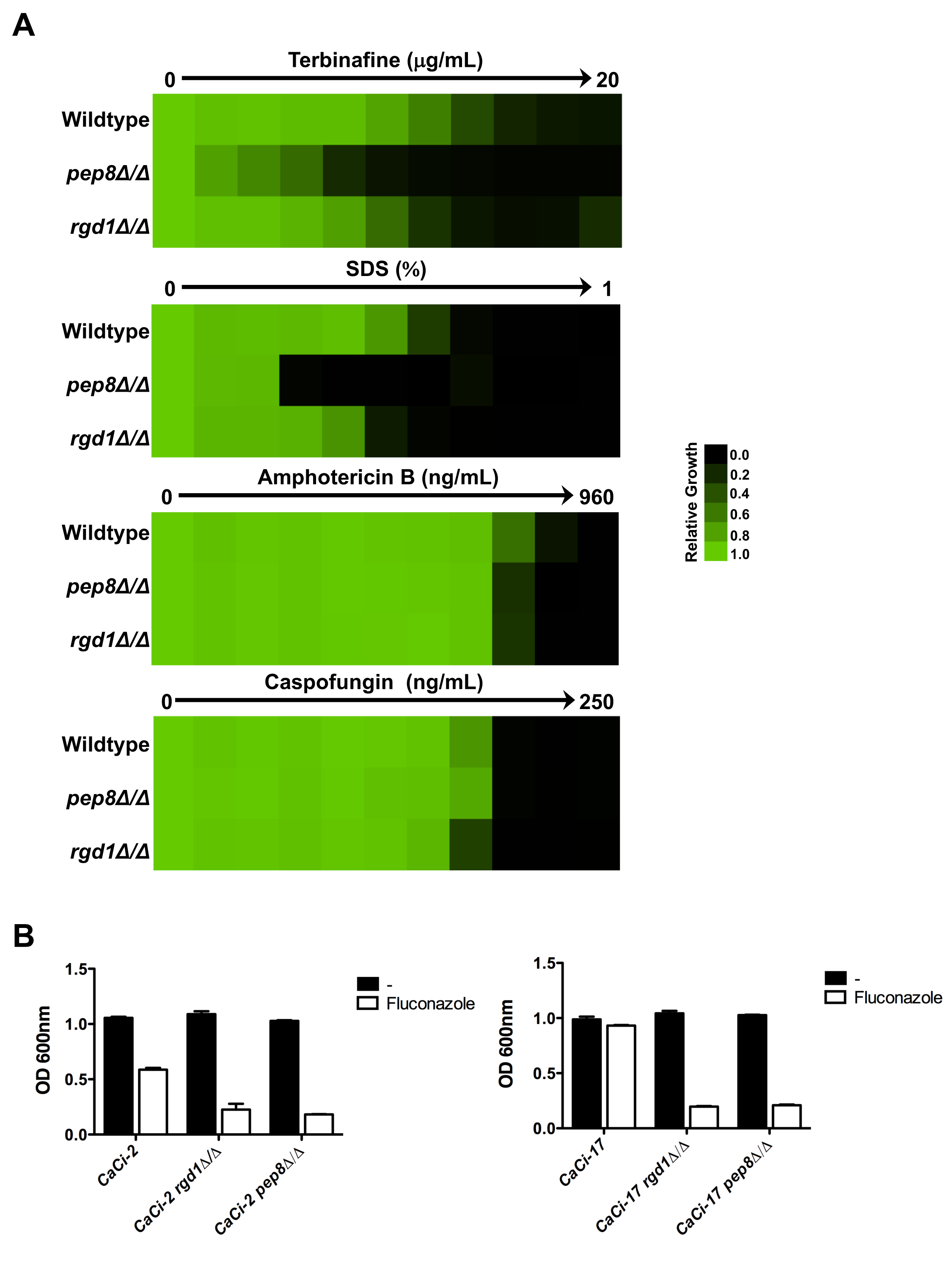

Supplement: S1 Fig — A) Microbroth dilution minimum inhibitory concentration (MIC) assays of wild type, rgd1Δ/Δ and pep8Δ/Δ mutants in response to diverse stresses. MIC assay was performed as described in Fig 1. Growth was measured after 24 hours. B) Histogram plots highlighting variation in OD600 values between technical duplicates for MIC plots shown in Fig 2D. Strains were grown in the absence or presence of 2 μg/mL of fluconazole (left plot) or 64 μg/mL (right plot). Optical densities were averaged for duplicate measurements and error bars represent standard deviation of duplicate measurements. MIC was performed in biological triplicate with similar results observed. (TIF) [file pgen.1007319.s006.tif]

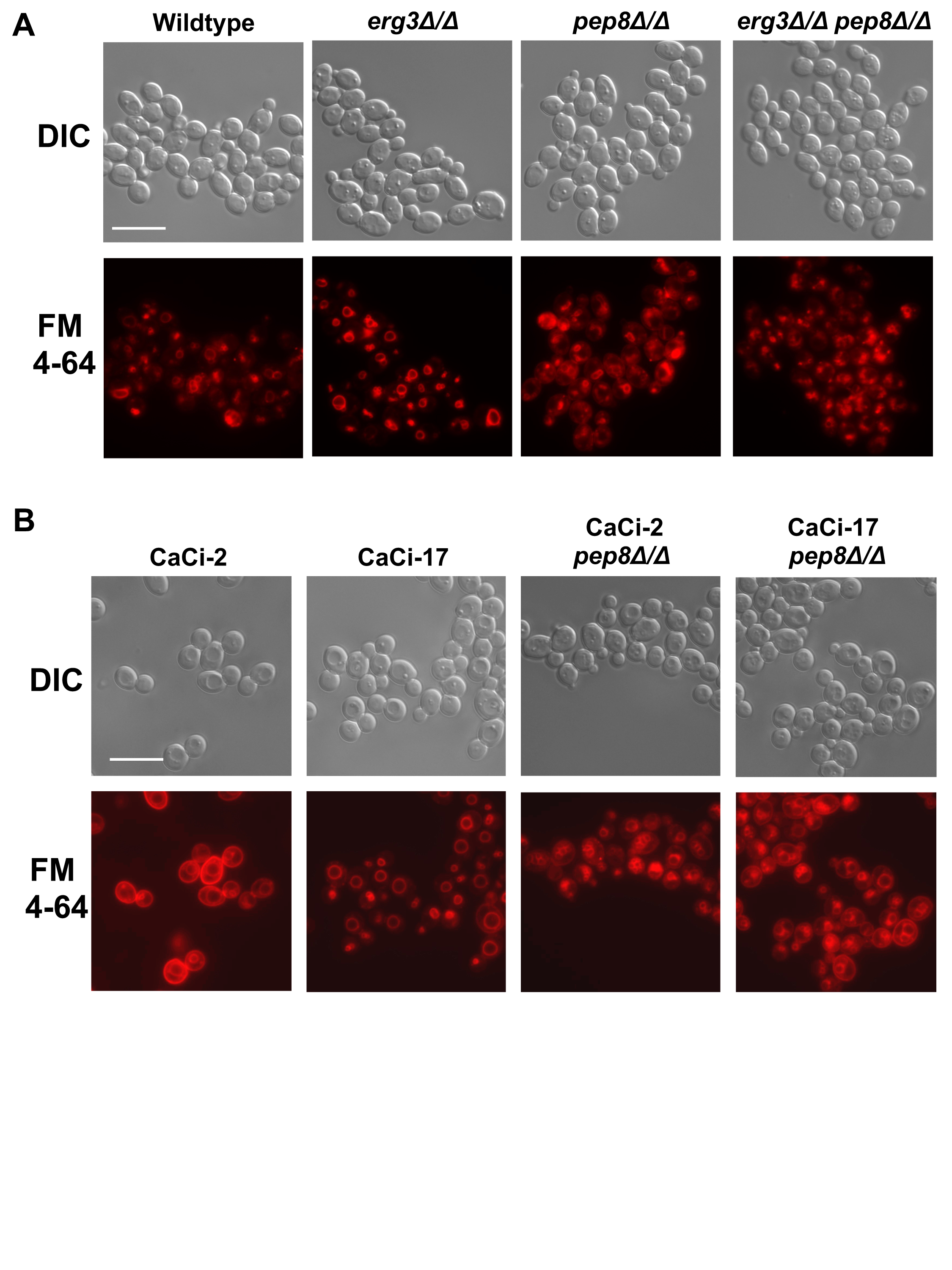

Supplement: S2 Fig — A) Strains of C. albicans were grown to log phase prior to staining with membrane dye FM4-64. Images were captured using differential interference contrast (DIC) microscopy and fluorescence microscopy with a TRITC/DsRED filter set on a Zeiss Axio Observer.Z1 (Carl Zeiss) using 100x magnification. Scale bar represents 10 μm. B) Strains of C. albicans were grown to log-phase prior to staining with membrane dye FM4-64. Images were captured using differential interference contrast (DIC) microscopy and fluorescence microscopy with a TRITC/DsRED filter set on a Zeiss Axio Observer.Z1 (Carl Zeiss) using 100x magnification. Scale bar represents 10 μm. (TIF) [file pgen.1007319.s007.tif]

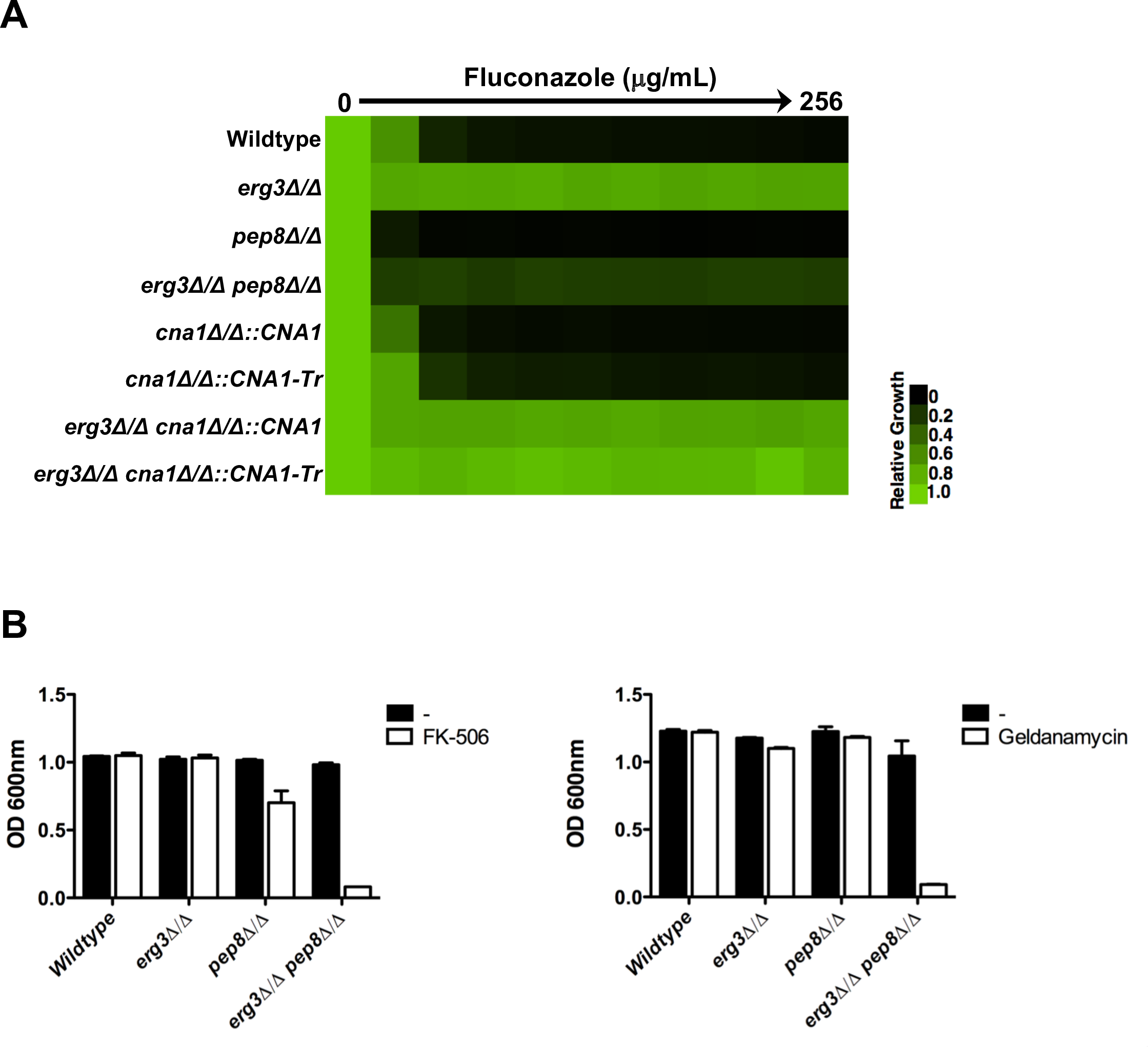

Supplement: S3 Fig — A) Hyperactive calcineurin does not abrogate erg3-mediated azole resistance. Resistance mediated by loss of ERG3 is maintained in a strain harbouring a hyperactive calcineurin allele (CNA1-Tr). MIC was performed as described in Fig 1. Growth was measured after 24 hours. B) Histogram plots highlighting variation in OD600 values between technical duplicates for MIC plots shown in Fig 3C. Strains were grown in the absence or presence of FK-506 (0.78 μM) or Geldanamycin (0.78 μM). Optical densities were averaged for duplicate measurements and error bars represent standard deviation of duplicate measurements. MIC was performed in biological triplicate with similar results observed. (TIF) [file pgen.1007319.s008.tif]

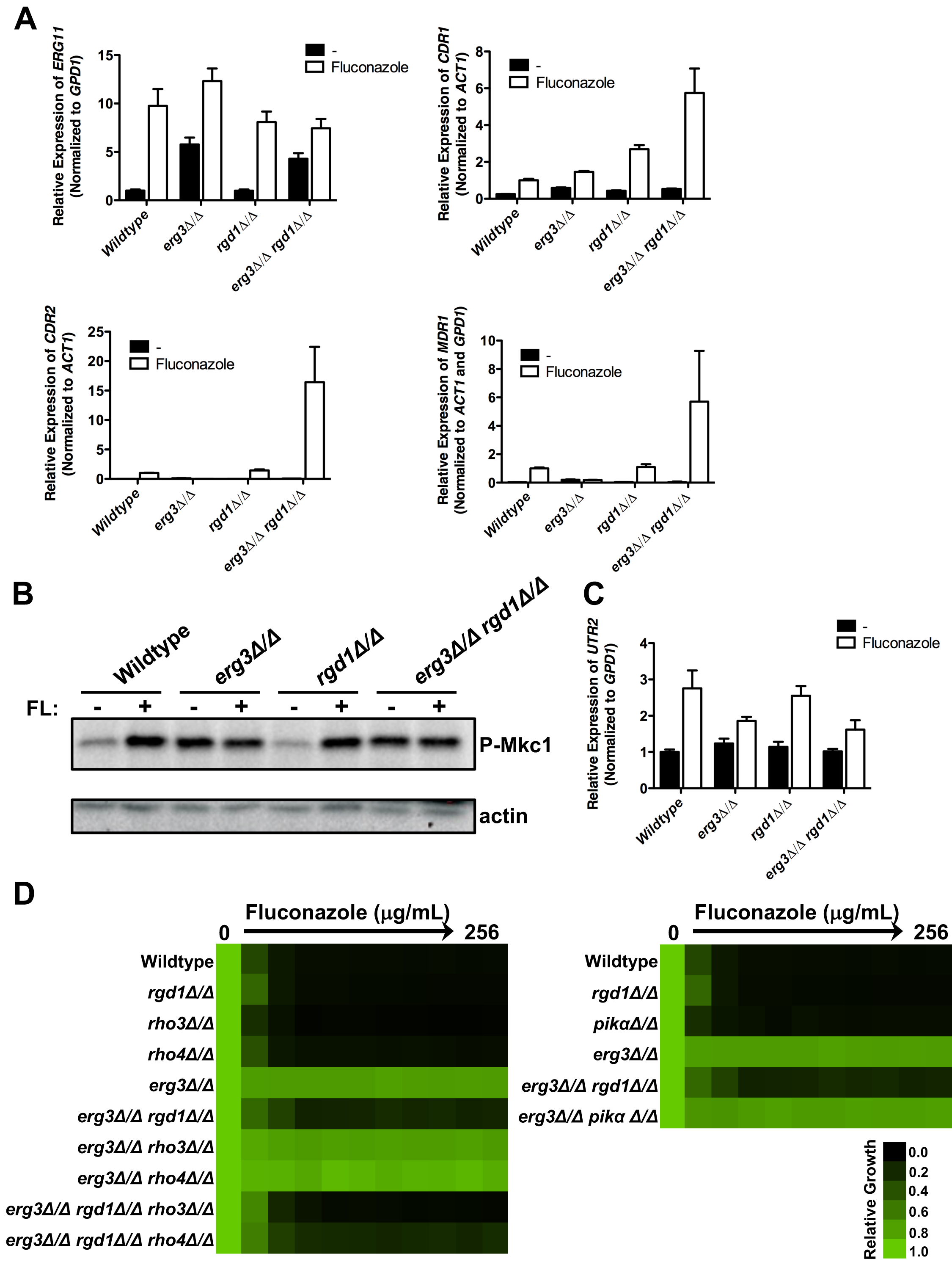

Supplement: S4 Fig — A) Deletion of RGD1 does not reduce expression of the azole target gene ERG11, nor the expression of efflux transporters CDR1, CDR2, or MDR1. Strains were grown in in YPD (-) or YPD with 16 μg/mL Fluconazole. Transcript levels were monitored by qRT-PCR and normalized to GPD1 or ACT1 as indicated. Error bars represent standard error of the mean for triplicate samples. B) Deletion of ERG3 leads to activation of the cell wall integrity signaling under basal conditions, but deletion of RGD1 does not block activation of cell wall integrity under basal conditions or in response to fluconazole (FL). Strains were left untreated or treated with 8 μg/mL FL for 40 minutes, as indicated. Phosphorylated Mkc1 (P-Mkc1) was monitored by Western blot and detected with an α-p44/42 antibody. Actin was detected with an α-β-actin antibody as a loading control. C) Deletion of RGD1 does not reduce expression of calcineurin-dependent transcript, UTR2. Transcript levels were monitored by qRT-PCR and normalized to GPD1. Error bars represent standard error of the mean for triplicate samples. D) Deletion of putative C. albicans RGD1 physical interactors, identified based on reports in S. cerevisiae, does not abrogate erg3-mediated resistance. MIC assay was performed as described in Fig 1. Growth was measured after 24 hours. (TIF) [file pgen.1007319.s009.tif]

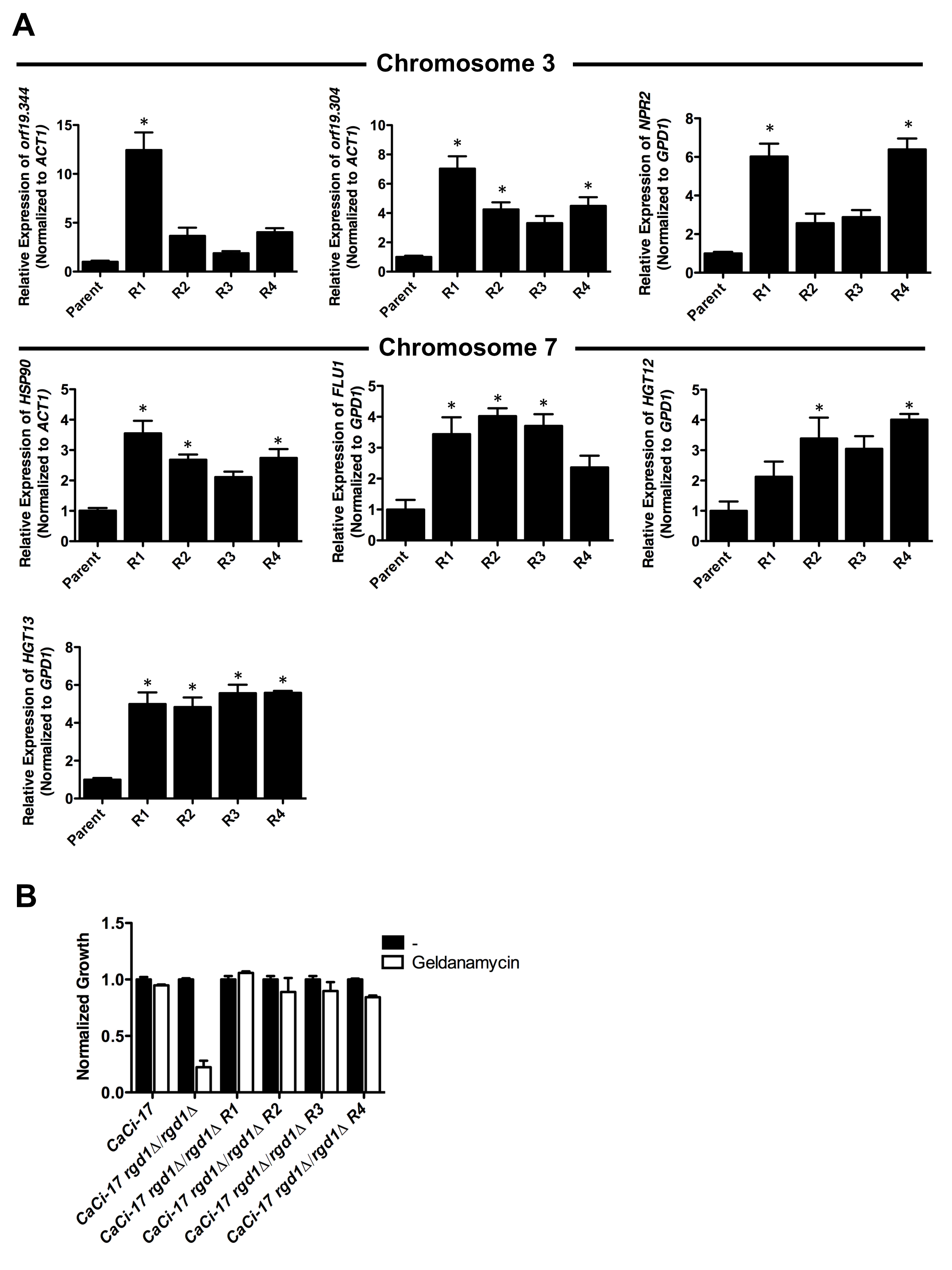

Supplement: S5 Fig — A) Azole-resistant isolates (R1-R4) have increased expression of HSP90, orf19.304, and orf19.344, FLU1, NPR2, HGT12, and HGT13 relative to the evolved parent. Transcript levels were monitored by qRT-PCR and normalized to GPD1 or ACT1 as indicated. Error bars represent standard error of the mean for triplicate samples. Expression levels of evolved strains were compared to the parental strain using a one-way ANOVA with Bonferroni post-test. Asterisk indicates significant difference in transcript level relative to the parental strain (* P<0.05). B) Spontaneous mutants show enhanced resistance to the Hsp90 inhibitor geldanamycin (GdA). Strains were grown in YPD medium in the absence (-) and presence of 25 μM GdA. Growth was measured by absorbance at 600 nm after 48 hours at 30°C and normalized to growth in the absence of inhibitor. Optical densities were averaged for duplicate measurements and error bars represent standard deviation of duplicate measurements. (TIF) [file pgen.1007319.s010.tif]

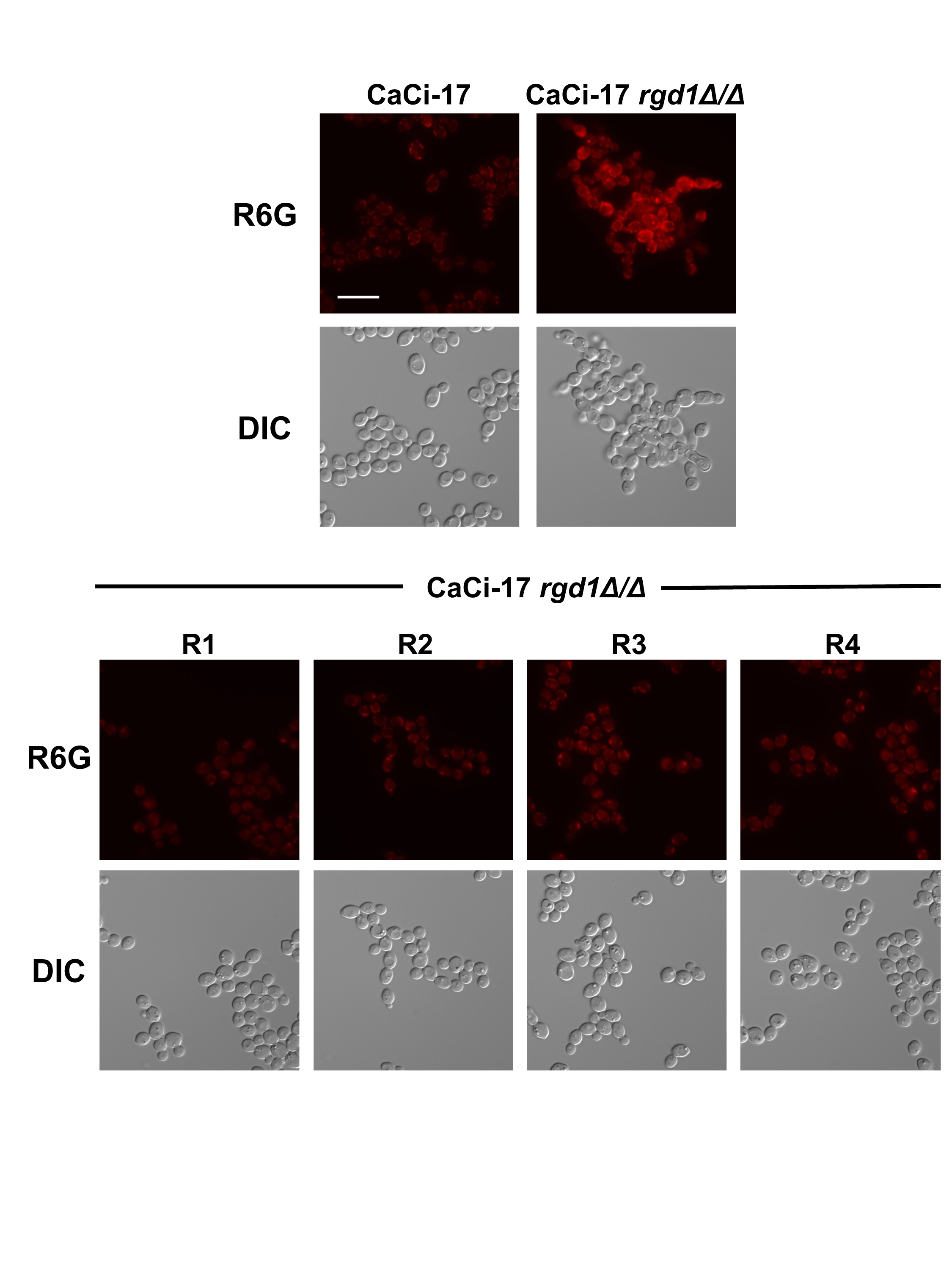

Supplement: S6 Fig — Deletion of RGD1 increases rhodamine-6G accumulation in a C. albicans clinical isolate (CaCi-17), which correlates with enhanced sensitivity to azoles. Selection of four azole-resistant CaCi-17 rgd1Δ/rgd1Δ lineages (R1-R4) results in decreased accumulation of rhodamine 6G. Scale bar represents 10 μm. Assay was performed in biological duplicates. (TIF) [file pgen.1007319.s011.tif]
